# Anlotinib enhances the efficacy of KRAS-G12C inhibitors through c-Myc/ORC2 axis inhibition in non-small cell lung cancer

**DOI:** 10.1038/s41419-025-07687-w

**Published:** 2025-05-02

**Authors:** Hongyu Liu, Chao Zhou, Jun Lu, Yuqing Liu, Peichen Zou, Liang Zhu, Huimin Lei, Baohui Han

**Affiliations:** 1 Department of Respiratory and Critical Care Medicine, Shanghai Chest Hospital, Shanghai Jiao Tong University School of Medicine, Shanghai, China; 2 Department of Pharmacology and Chemical Biology, College of Basic Medical Sciences, Shanghai Jiao Tong University School of Medicine, Shanghai, China

**Keywords:** Non-small-cell lung cancer, Cancer therapeutic resistance, Targeted therapies

## Abstract

Non-small cell lung cancer (NSCLC) remains a leading cause of cancer-related mortality globally, with KRAS mutations present in approximately 20–25% of cases. The KRAS-G12C mutation, occurring in approximately 14% of lung adenocarcinomas, has emerged as a critical target for precision medicine strategies. While KRAS-G12C inhibitors, including sotorasib and adagrasib, have shown promise in clinical trials, their efficacy is limited by primary and acquired resistance mechanisms. This study explored the potential of combining anlotinib, a multi-target tyrosine kinase inhibitor, with KRAS-G12C inhibitors to overcome these resistance challenges in NSCLC treatment. Our results demonstrated that anlotinib improved the sensitivity to KRAS-G12C inhibitors in primary and acquired resistance settings, both in vitro and in vivo. Mechanistically, the combination therapy inhibited c-Myc/ORC2 signaling, leading to cell cycle arrest and apoptosis. These findings suggest that the combination of anlotinib and KRAS-G12C inhibitors represents a promising novel therapeutic approach for KRAS-G12C-mutant NSCLC.

## Introduction

Non-small cell lung cancer (NSCLC) remains a significant global health burden, accounting for a substantial portion of cancer-related deaths worldwide [1]. One of the most promising developments in NSCLC treatment is the targeting of KRAS mutations, which occur in 20–25% of cases and have historically been regarded as “undruggable”, particularly the KRAS-G12C mutation [2–4]. The KRAS-G12C mutation is present in approximately 14% of lung adenocarcinomas, establishing it as a critical target for precision medicine approaches [5, 6]. The development of KRAS-G12C inhibitors (KRAS-G12Ci), such as sotorasib and adagrasib, have shown promising results in clinical trials, demonstrating moderate benefits for previously treated KRAS-G12C mutant NSCLC patients, although overall survival remains unimproved [7, 8].

The efficacy of KRAS-G12Ci is constrained by both primary and acquired resistance mechanisms [9]. Primary resistance, observed in about 60% of patients, may be attributed to a lack of dependency on KRAS signaling [10]. Acquired resistance often involves adaptive non-mutational reprogramming and the feedback reactivation of compensatory signaling pathways [10–12]. To address these challenges, combination therapies targeting multiple pathways have been proposed, which involves the combination of KRAS-G12C inhibitors with agents that target complementary pathways or resistance mechanisms [12, 13]. In this context, anlotinib has emerged as a promising candidate for combination therapy.

Anlotinib is a novel multi-target tyrosine kinase inhibitor that has demonstrated significant antitumor activity across various cancer types [14]. It primarily targets vascular endothelial growth factor receptor, fibroblast growth factor receptor, platelet-derived growth factor receptor, and c-Kit [14, 15]. Anlotinib has demonstrated efficacy in clinical trials for NSCLC, particularly in patients who have progressed following standard therapies [16]. Its capacity to inhibit multiple pathways involved in tumor growth and angiogenesis renders it an attractive option for combination strategies.

In this study, we evaluated the antitumor effects of combining anlotinib with KRAS-G12Ci. Our results reveal that this combination inhibits c-Myc/origin recognition complex subunit 2 (ORC2) signaling, induces cell cycle arrest and apoptosis, and exerts potent antitumor effects in vitro and in vivo under both primary and acquired KRAS-G12Ci resistance conditions. These findings establish the combination therapy as a promising clinical strategy for overcoming KRAS-G12Ci resistance.

## Methods

### Cell lines and cell culture

Six NSCLC cell lines harboring KRAS-G12C mutations (H2122, H2030, H358, H23, SW1573, and Calu-1) were used in this study. H2122 and H2030 were purchased from Zhong Qiao Xin Zhou Biotechnology (Shanghai, China); H358 and H23 were purchased from the Cell Bank of the Chinese Academy of Sciences (Shanghai, China); and SW1573 and Calu-1 were acquired from the Central Laboratory of Shanghai Chest Hospital. Cell lines were authenticated by short tandem repeat (STR) profiling. Cells were maintained in RPMI 1640 medium (Gibco) supplemented with 10% fetal bovine serum (GeminiBio), 1% penicillin-streptomycin (BasalMedia), and 1% GlutaMax (BasalMedia) under standard culture conditions (37 °C, 5% CO_2_ in a humidified atmosphere).

### Reagents and antibodies

Anlotinib was provided by Chia Tai Tianqing Pharmaceutical Group Co., Ltd (Nanjing, China). Sotorasib, adagrasib and MG132 were purchased from MedChemExpress (New Jersey, USA). Stock solutions were prepared by dissolving sotorasib, adagrasib and MG132 in dimethyl sulfoxide (DMSO), while anlotinib was dissolved in sterile water. All compounds were stored at −20 °C and diluted to working concentrations immediately before use. The primary antibodies used in this study were as follows: Cyclin B1 (Proteintech, 55004-1-AP), Cyclin E1 (Proteintech, 11554-1-AP), Cyclin D1 (Proteintech, 60186-1-Ig), CDK2 (Proteintech, 60312-1-Ig), GAPDH (Proteintech, HRP-60004), Mcl-1 (Proteintech, 16225-1-AP), Bcl-2 (Proteintech, 12789-1-AP), Bax (ABclonal, A19684), Bid (ABclonal, A23234), p-Erk1/2 (Cell Signaling Technology, 4370), Erk1/2 (Cell Signaling Technology, 4695), p-Akt (Cell Signaling Technology, 4060), Akt (Cell Signaling Technology, 4691), β-actin (ABclonal, AC006), c-Myc (Cell Signaling Technology, 5605), COPS5 (ABclonal, A4087), eIF4E (ABclonal, A2162), ORC2 (Abcam, A15697).

### Cell viability and cell growth assay in 2D culture

Cell viability was assessed using the Cell Counting Kit-8 (CCK-8; Dojindo Laboratories). Cells were seeded in 96-well plates at a density of 3–5 × 10^3^ cells per well and treated with indicated compounds for 72 h. Following treatment, cells were incubated with CCK-8 reagent for 1 and 2 h at 37 °C. Absorbance was measured at 450 nm using a microplate reader. Cell growth was monitored using the IncuCyte Zoom Live-Cell Analysis System (Essen BioScience).

### Synergism analysis

Drug combination effects were quantified using mutiple methods. For Chou-Talalay method, combination index (CI) values were calculated using CompuSyn software (Version 1.0). CI values were interpreted as follows: CI < 1.0 indicating synergism, CI = 1.0 indicating additivity, and CI > 1.0 indicating antagonism. For ZIP, HSA, Bliss, and Loewe score analysis, the Synergyfinder R package (Zheng S, Wang W, Aldahdooh J 2022) was used according to user tutorial. Synergy score was interpreted as follows: Synergy score < −10 indicating antagonism; −10 < Synergy score < 10 indicating additivity, and Synergy score > 10 indicating synergism.

### Colony formation assay

For assessment of long-term cell survival and proliferation capacity, cells were seeded at low density (1000 cells/well in six-well plates or 200 cells/well in 96-well plates) and cultured for 14 days with regular medium changes every 3 days. Following the culture period, medium was aspirated, and colonies were fixed with 4% polyoxymethylene for 15 min at room temperature. Fixed colonies were stained with 0.1% crystal violet solution for 30 min. After gentle washing with PBS to remove excess stain, plates were air-dried and photographed. Colony number and size were quantified using ImageJ software (NIH, v1.54).

### Wound healing assay

Cells were seeded into 96-well plates at ~80%–90% confluency overnight, and scratch was then made with IncuCyte Wound Maker 96-Tool (Essen Bioscience). After removing detached cells, fresh medium containing indicated compounds was replenished. Wound confluence was monitored and analyzed using IncuCyte Zoom Live-Cell Analysis System.

### Cell cycle and apoptosis analysis

For cell cycle analysis, cells were incubated with 10 μM EdU in RPMI 1640 for 3 h using the EdU Cell Proliferation Kit with Alexa Fluor 647 (CX004, Epizyme). Following fixation with polyoxymethylene and permeabilization with 0.5% Triton X-100, cells were stained with EdU-Alexa Fluor 647 and propidium iodide (PI) using the Cell Cycle Staining Kit (CCS012, MultiSciences). Analysis was performed using an Attune NxT Flow Cytometer (Thermo Fisher Scientific). For apoptosis detection, cells were double-stained with Annexin V-FITC and PI using the Annexin V-FITC/PI Apoptosis Kit (AT101, MultiSciences) according to manufacturer’s instructions. Early apoptotic cells were defined as Annexin V-positive/PI-negative, while late apoptotic cells were Annexin V-positive/PI-positive. Results were analyzed using Flowjo software (FlowJo LLC, v10.8.1).

### Western blotting

Total protein was extracted using RIPA lysis buffer supplemented with protease and phosphatase inhibitors. Protein concentrations were determined using the bicinchoninic acid assay. Equal amounts of protein were separated by 10% SDS-PAGE and transferred to PVDF membranes. Membranes were blocked with 5% non-fat milk in TBST for 1 h at room temperature, followed by overnight incubation with primary antibodies at 4 °C. Membranes were then incubated with HRP-conjugated secondary antibodies for 1 h at room temperature. Protein bands were visualized using an electrochemiluminescence imager. Results were analyzed in Image Studio software (LI-COR).

### Transcriptome sequencing (RNA-seq)

The total RNA of three replicates was isolated using TRIzol Reagent (Invitrogen). Library construction and paired-end sequencing were performed on an Illumina Novaseq 6000 platform. Raw sequencing data were quality-controlled and mapped to the human reference genome (GRCh38). Differential expression analysis was conducted using the DESeq2 R package (Love MI, Huber W, Anders S 2014), and pathway enrichment analysis was performed using gene set enrichment analysis (GSEA) software (UC San Diego and Broad Institute, v4.1.0). Differentially expressed genes were defined as |log2FC| > 2 and adjusted *p*-val < 0.05.

### RNA extraction and quantitative PCR

Total RNA of was extracted using FastPure Cell/Tissue Total RNA Isolation Kit (RC112-01, Vazyme) following manufacturer’s protocol. RNA quality and concentration were assessed using NanoDrop 2000 spectrophotometer (Thermo Fisher Scientific). First-strand cDNA was synthesized using HiScript IV All-in-One Ultra RT SuperMix (R433-01, Vazyme). Real-time PCR was performed using Taq Pro Universal SYBR qPCR Master Mix (Q712-02, Vazyme) on a LightCycler 480 Instrument II (Roche). The sequences from 5ʹ to 3ʹ of primers used in this study were as follows:

**Table:** 

Actin	forward CACCATTGGCAATGAGCGGTTC
	reverse AGGTCTTTGCGGATGTCCACGT
SNRPD2	forward AGTCAAGAACAATACCCAAGTGC
	reverse ATGTTGCAGTGCCTATCGAAG
RPL22	forward AAAGTGAACGGAAAAGCTGGG
	reverse TCACGGTGATCTTGCTCTTGC
DEK	forward AACTGCTTTACAACAGGCCAG
	reverse ATGGTTTGCCAGAAGGCTTTG
EIF4E	forward GAAACCACCCCTACTCCTAATCC
	reverse AGAGTGCCCATCTGTTCTGTA
CBX3	forward TAGATCGACGTGTAGTGAATGGG
	reverse TGTCTGTGGCACCAATTATTCTT
COPS5	forward TGGGTCTGATGCTAGGAAAGG
	reverse CTATGATACCACCCGATTGCATT
ORC2	forward GCTTCAGACAAGGTTCAACCG
	reverse CTGTGCAACCCCTTCATCATC
SSB	forward AATTTGCCACGGGACAAGTTT
	reverse TGTTGTTAGACGGTTCAACCTG
UBE2E1	forward TCCGTGTATGAGGGTGGTGTA
	reverse CGAAATGTAACCTTTGGAGGCTT
AP3S1	forward TACCAGCCCTACAGTGAAGATAC
	reverse ATCAGTTTGTTGTCAGATCCTCC

### Plasmid and siRNA transfection

Cells were seeded in six-well plates at 40–50% confluency and cultured overnight. For plasmid transfection, DNA was mixed with Lipofectamine 3000 and P3000 reagent (Invitrogen) according to manufacturer’s protocol. Plasmid for human MYC (NM_002467.6) was constructed by GeneChem (Shanghai, China). For small interfering RNA (siRNA) transfection, oligonucleotides (20 μM) were mixed with Lipofectamine 3000. After 72 h of transfection, cells were reseeded into six-well or 96-well plates for subsequent experiments. Transfection efficiency was validated by western blot analysis.

The ORC2 siRNA sequences from 5ʹ to 3ʹ were as follows:

si-ORC2 #1: GGUUCAACAUUGUGCUUUATT

si-ORC2 #2: CCUGUUGAUAAUGGAACAUTT

### Cell-derived xenograft (CDX) assay and in vivo treatment

Five-week-old nu/nu female athymic mice were subcutaneously injected with 1 × 10^7^ cells suspended in 100 μl PBS into the flank region. Tumor dimensions were measured every 3 and 4 days using digital calipers, and tumor volume was calculated using the formula: volume = length × width^2^ × 0.5. When tumors reached approximately 200 mm^3^, mice were randomized into four treatment groups. For H23 CDX, mice were treated with vehicle, sotorasib (60 mg/kg, daily), anlotinib (2 mg/kg, daily) or combination (sotorasib plus anlotinib) through intragastric administration. For H2122SR CDX, mice were treatment with vehicle, sotorasib (100 mg/kg, daily), anlotinib (2 mg/kg, daily) or combination (sotorasib plus anlotinib). Sotorasib was dissolved in 1% Tween 80, 2% HPMC and 97% water, and anlotinib was dissolved in water. Mice were euthanized when tumors exceeded 1500 mm^3^ or at experiment endpoint. Tumors were harvested, weighed, photographed, and fixed in polyoxymethylene for 24 h for subsequent analysis. All procedures were performed with the approval of the Institutional Animal Care and Use Committee of Shanghai Jiao Tong University School of Medicine.

### Immunohistochemistry

Fixed tumor tissues were paraffin-embedded and sectioned. Tissue sections underwent antigen retrieval and endogenous peroxidase blockade, followed by overnight incubation at 4 °C with primary antibodies against Ki-67, c-Myc, or ORC2. Sections were scanned using an Ocus 20 Microscope Slide Scanner (Grundium) and analyzed using QuPath software (v0.5.1) for quantitative assessment.

### Tissue microarray (TMA) analysis

A tissue microarray (TMA) containing samples from 80 NSCLC patients was obtained from Changsha Yaxiang Biotechnology Co., LTD, along with corresponding clinical characteristics and survival data. The study was approved by the company’s Life Sciences Ethics Committee (query code: PZEU12C6DYBMQT). TMA sections were immunohistochemically stained for c-Myc and ORC2. Stained sections were analyzed using QuPath software (v0.5.1) for quantitative assessment of protein expression.

### Statistics

Quantitative data from in vitro and in vivo assays are presented as mean ± standard error of the mean (SEM). Statistical differences between groups were determined using two-tailed Student’s *t*-test for comparisons between two groups, or one-way or two-way analysis of variance (ANOVA) followed by appropriate post-hoc tests for multiple comparisons. Pearson analysis was performed in correlation analysis. All statistical analyses and graphical representations were performed using GraphPad Prism 10 software. For clinical data analysis, survival curves were generated using the Kaplan–Meier method. Statistical differences in survival were assessed using the log-rank test. *P*-values < 0.05 were considered statistically significant.

## Results

### Anlotinib enhances the sensitivity to KRAS-G12Ci of primary and acquired resistant cells in vitro

To explore the effects of anlotinib combined with KRAS-G12Ci in KRAS-G12Ci resistance settings, we first investigated the inhibitory efficacy of sotorasib across different NSCLC cell lines harboring KRAS-G12C mutations. The sensitivity to sotorasib varied significantly among cell lines: H358, H2122, and H2030 showed high sensitivity, while H23, Calu-1, and SW1573 maintained viability even at high sotorasib concentrations (Fig. S1A). Based on these results, H23, Calu-1, and SW1573 were classified as primary resistant cell lines.

To study acquired resistance, we established acquired resistant cell lines (H2122SR and H2030SR) by exposing H2122 and H2030 cells to increasing concentrations of sotorasib (Fig. S1B). Resistance was confirmed through cell viability assays (Fig. S1C, D). Notably, both primary and acquired resistant cell lines maintained sensitivity to anlotinib (Fig. S1E), and H2122SR and H2030SR were slightly more sensitive to anlotinib (Fig. S1F).

**Fig. 1:**
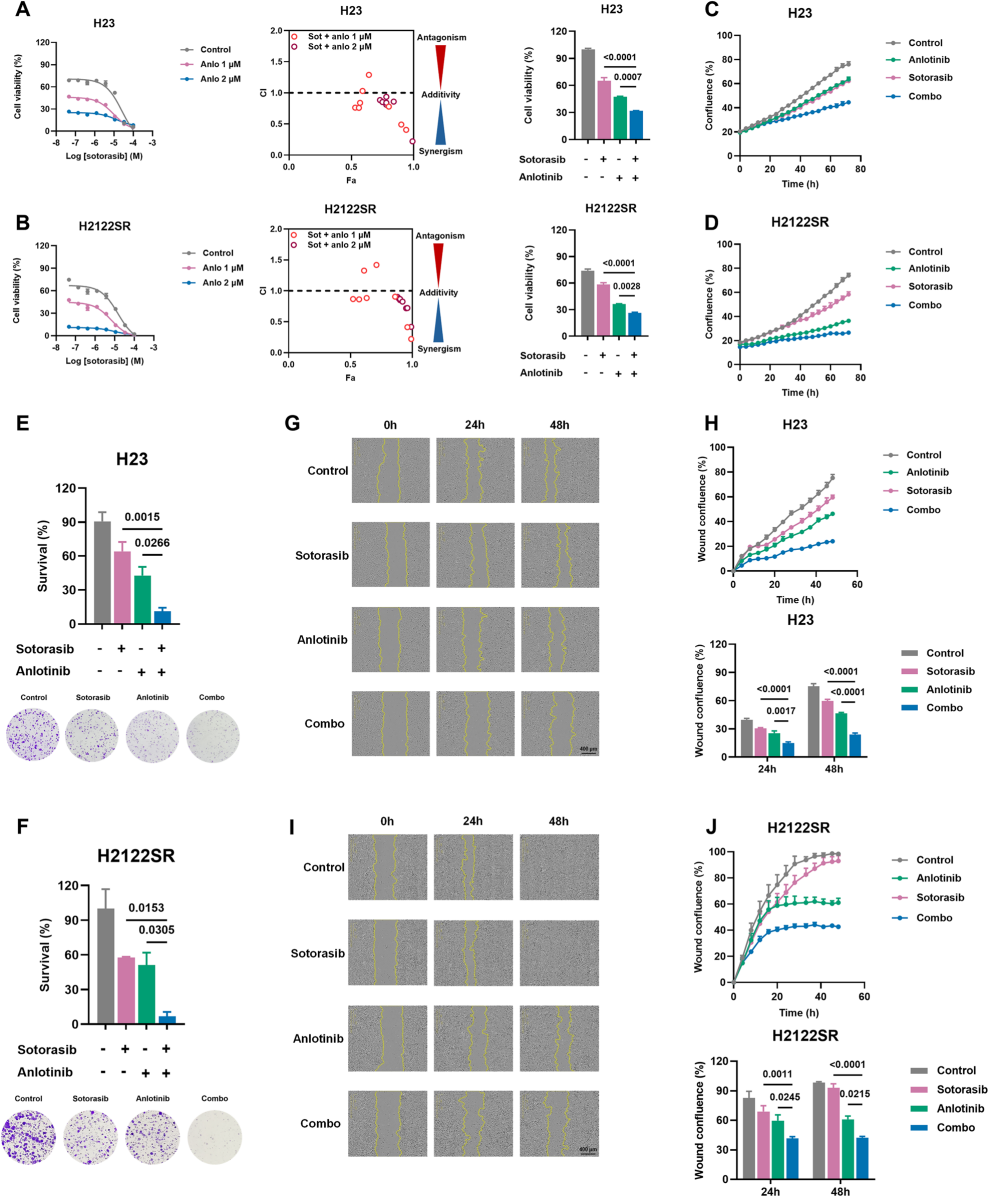
Anlotinib enhances the sensitivity to KRAS-G12Ci of primary and acquired resistant cells in vitro. **A**, **B** Cell viability and combination index of anlotinib combined with sotorasib treated H23 (**A**) and H2122SR (**B**). Results are shown as mean ± SEM. Statistical differences are determined using one-way ANOVA with Tukey’s multiple comparisons test. Bold: *p* < 0.05. *n* = 3 per group. CI: combination index, Fa fraction affected, Sot sotorasib, Anlo anlotinib. **C**, **D** Cell growth of H23 (**C**) and H2122SR (**D**) treated with anlotinib (2 μM) plus sotorasib (1 μM) monitored using IncuCyte. Results are shown as mean ± SEM. Bold: *p* < 0.05. *n* = 3 per group. Combo: anlotinib plus sotorasib. **E**, **F** Colony formation assays of H23 (**E**) and H2122SR (**F**) treated with anlotinib (1 μM) plus sotorasib (1 μM) for 14 days. Results are shown as mean ± SEM. Statistical differences are determined using one-way ANOVA with Tukey’s multiple comparisons test. Bold: *p* < 0.05. *n* = 3 per group. Combo: anlotinib plus sotorasib. **G–J** Wound healing assays of H23 (**G**, **H**) and H2122SR (**I**, **J**) treated with anlotinib (2 μM) plus sotorasib (1 μM) for 48 h. Results are shown as mean ± SEM. Statistical differences are determined using two-way ANOVA with Tukey’s multiple comparisons test. Bold: *p* < 0.05. *n* = 3 per group. Combo: anlotinib plus sotorasib.

Cell viability assays and synergy analysis revealed that anlotinib dose-dependently enhanced sotorasib’s effects in all five resistant cell lines (H23, Calu-1, SW1573, H2122SR, H2030SR), with synergistic effects observed across most concentration combinations (Figs. 1A, B, S2A–E, and Supplementary Table 1). Similar effects were observed when anlotinib was combined with another KRAS-G12C inhibitor, adagrasib (Fig. S2I, J). The combination significantly suppressed cell growth compared to either agent alone (Figs. 1C, D and S2F–H). Colony formation assays demonstrated that anlotinib enhanced sotorasib’s inhibition of long-term cell survival (Figs. 1E, F and S2K, L). Additionally, wound healing assays showed the combination more effectively inhibited cell migration in resistant cell lines (Figs. 1G–J and S2M–P). These findings collectively demonstrate that anlotinib enhances KRAS-G12Ci’s antitumor effects in both primary and acquired resistant settings in vitro.

### Anlotinib combined with KRAS-G12Ci induces cell cycle arrest and apoptosis in primary and acquired resistant cells

**Fig. 2:**
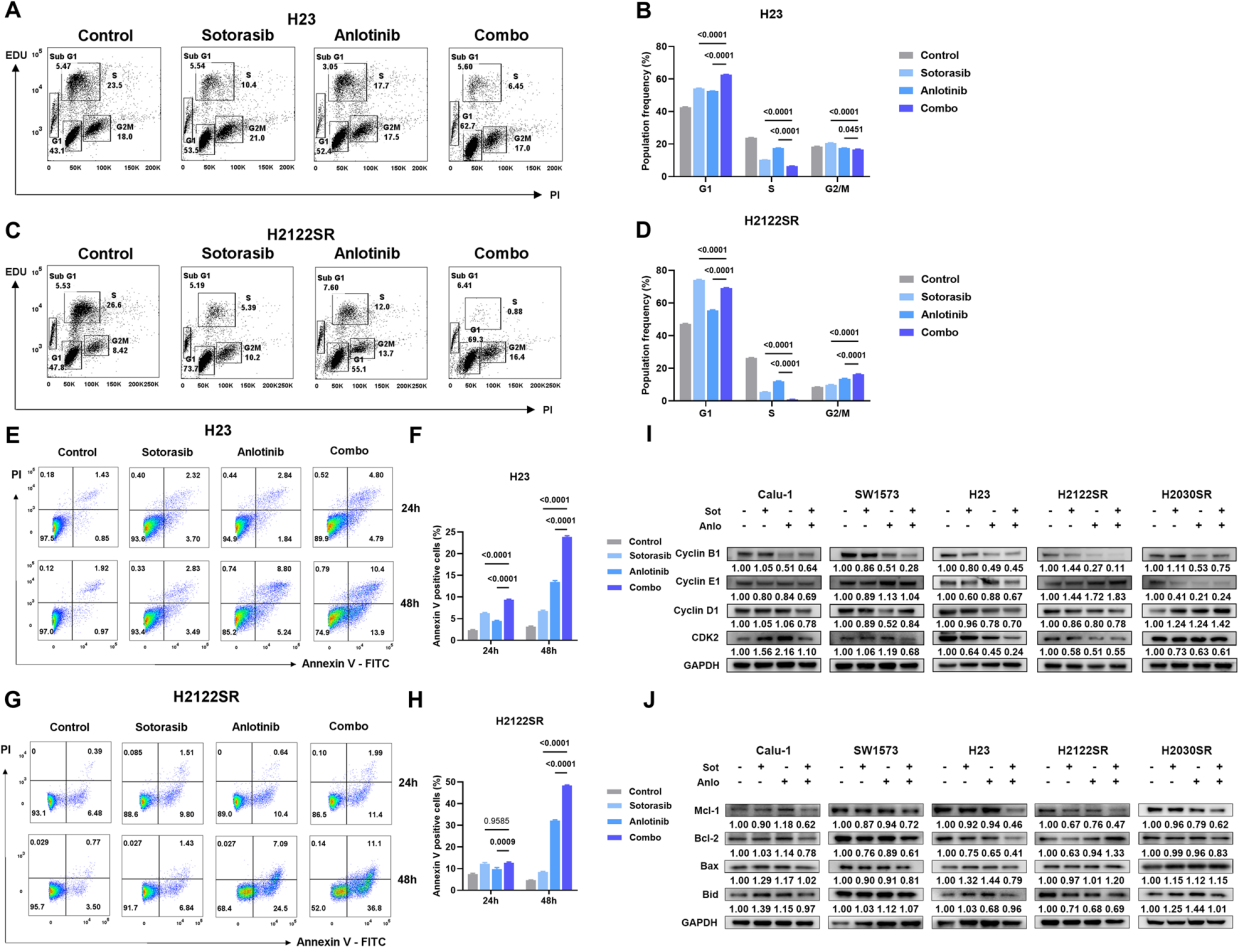
Anlotinib combined with KRAS-G12Ci induces cell cycle arrest and apoptosis in primary and acquired resistant cells. **A–D** EdU / PI staining assays of H23 (**A**, **B**) and H2122SR (**C**, **D**) treated with anlotinib (2 μM) plus sotorasib (1 μM) for 24 h. Results are shown as mean ± SEM. Statistical differences are determined using one-way ANOVA with Tukey’s multiple comparisons test. Bold: *p* < 0.05. *n* = 3 per group. Combo: anlotinib plus sotorasib. **E–H** Cell apoptosis assays of H23 (**E**, **F**) and H2122SR (**G**, **H**) treated with anlotinib (2 μM) plus sotorasib (1 μM) for 24 and 48 h. Results are shown as mean ± SEM. Statistical differences are determined using two-way ANOVA with Tukey’s multiple comparisons test. Bold: *p* < 0.05. *n* = 3 per group. Combo: anlotinib plus sotorasib. **I** Expression of Cyclin B1, Cyclin E1, Cyclin D1, and CDK2 in five cell lines (Calu-1, SW1573, H23, H2122SR and H2030SR) treated with anlotinib (2 μM) plus sotorasib (1 μM) for 24 h detected by western blotting. Sot sotorasib, Anlo anlotinib. **J** Expression of Mcl-1, Bcl-2, Bax, and Bid in five cell lines (Calu-1, SW1573, H23, H2122SR and H2030SR) treated with anlotinib (2 μM) plus sotorasib (1 μM) for 24 h detected by western blotting. Sot sotorasib, Anlo anlotinib.

To further characterize the mechanisms underlying the combination treatment’s efficacy, we examined its effects on cell cycle progression and apoptosis in resistant NSCLC cell lines. Cell cycle analysis revealed distinct patterns of arrest between primary and acquired resistant lines. In primary resistant cell lines, the combination treatment induced significant G1 phase arrest, while acquired resistant lines showed both G1 and G2/M phase arrest. Both scenarios resulted in markedly reduced S phase populations compared to single-agent treatments (Figs. 2A–D and S3A, B). Western blot analysis confirmed that the combination treatment more effectively suppressed key cell cycle regulators, including Cyclin B1, Cyclin E1, Cyclin D1, and CDK2, though the pattern of inhibition varied among cell lines (Fig. 2I).

Time-course analysis of apoptosis demonstrated that the combination treatment induced significantly higher apoptosis rates compared to single agents, with effects increasing over time (Figs. 2E–H and S3C, D). Mechanistically, the enhanced apoptotic response correlated with suppression of anti-apoptotic proteins (Mcl-1 and Bcl-2) rather than changes in pro-apoptotic proteins (Bax and Bid) (Fig. 2J). These findings demonstrate that anlotinib combined with KRAS-G12Ci effectively overcomes both primary and acquired resistance through dual mechanisms: inducing cell cycle arrest with consequent S phase reduction and promoting robust apoptotic responses.

### The sensitization effect of anlotinib on resistant cells is mediated through inhibition of c-Myc

To elucidate the mechanism underlying the enhanced antitumor effects, we first investigated changes in classical RAS signaling in resistant cell lines. While sotorasib maintained its ability to inhibit p-Erk significantly, it showed minimal effects on p-Akt, and anlotinib did not produce additional effects on RAS signaling (Fig. S4A).

**Fig. 3:**
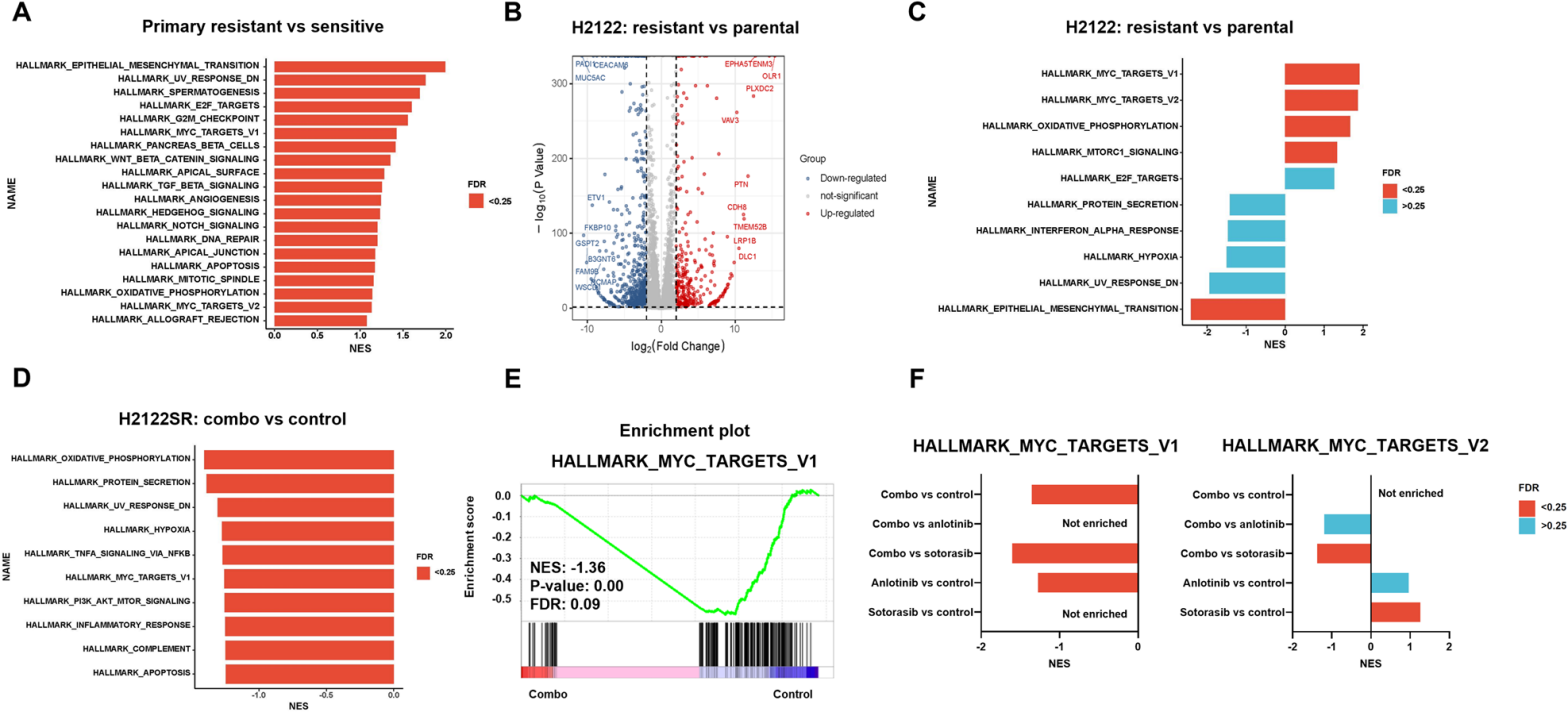
Transcriptome analysis revealed that MYC is the key regulator of the antitumor effects of anlotinib combined with KRAS-G12Ci. **A** Enriched hallmark pathways of primary resistant cell lines (Calu-1, SW1573, H23) compared with sensitive cell lines (H358, H2122, H2030). Normalized enrichment score (NES) is plotted. False discovery rate (FDR) < 0.25 is marked in red. Data are from CCLE datasets. **B** Volcano plot showing the transcriptional difference of H2122SR compared with H2122 parental cell line. Genes with log_2_FC > 2 and adjusted *p* value < 0.05 were marked in red. Genes with log_2_FC < −2 and adjusted *p* value < 0.05 were marked in blue. **C** Enriched hallmark pathways of H2122SR compared with H2122 parental cell line. Normalized enrichment score (NES) is plotted. FDR < 0.25 is marked in red. FDR > 0.25 is marked in blue. **D** Enriched hallmark pathways of H2122SR treated with anlotinib (2 μM) plus sotorasib (1 μM) for 24 h compared with vehicle. Normalized enrichment score (NES) is plotted. FDR < 0.25 is marked in red. Combo: anlotinib plus sotorasib. **E** GSEA enrichment plot of MYC targets V1 pathway of H2122SR treated with anlotinib (2 μM) plus sotorasib (1 μM) for 24 h compared with vehicle. **F** Barplot comparing NSE and FDR of MYC targets V1 and MYC targets V2 pathways enriched in different treatment groups: DMSO, anlotinib (2 μM), sotorasib (1 μM), and combo (anlotinib plus sotorasib) for 24 h. FDR < 0.25 is marked in red. FDR > 0.25 is marked in blue. Combo: anlotinib plus sotorasib.

Transcriptome analysis of primary sensitive versus resistant cell lines using the CCLE database [17] revealed multiple enriched malignancy-related pathways in resistant cells, including EMT, E2F targets, G2M checkpoint, and MYC targets (Figs. 3A and S4B). Similarly, GSEA of an adagrasib-resistant SW837 (KRAS-G12C mutant colorectal adenocarcinoma cell line) CDX model [18] also suggested that MYC targets were among top enriched pathways compared to treatment naïve tumors (Fig. S4C).

RNA-seq comparison between H2122 and its resistant counterpart H2122SR identified 902 upregulated and 1601 downregulated genes (Fig. 3B). GSEA analysis highlighted MYC targets V1, MYC targets V2, and oxidative phosphorylation as the top three significantly enriched pathways in H2122SR (Figs. 3C and S4D).

Transcriptome analysis of H2122SR cells after 24-h treatment with sotorasib, anlotinib, or their combination revealed enrichment of various pathways in combination-treated cells, including TNF-α signaling via NF-κB, MYC targets V1, and PI3K-Akt-mTOR signaling (Figs. 3D, E and S4E).

**Fig. 4:**
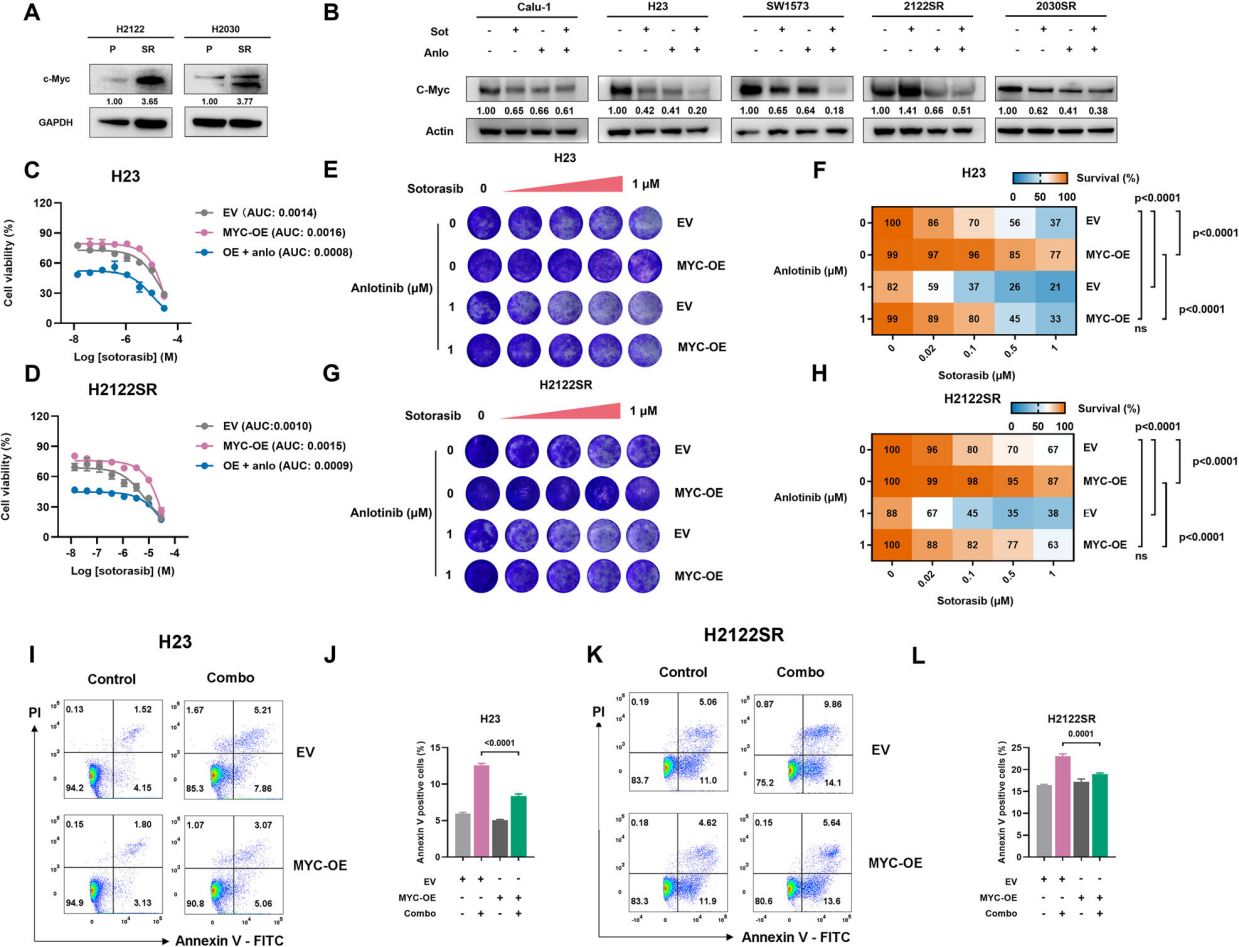
The sensitization effect of anlotinib on resistant cells is mediated through inhibition of c-Myc. **A**, **B** The expression of c-Myc in H2122SR and H2030SR compared with parental cell lines (**A**) and in five cell lines (Calu-1, SW1573, H23, H2122SR, and H2030SR) treated with anlotinib (2 μM) plus sotorasib (1 μM) for 24 h (**B**) detected by western blotting. Sot sotorasib, Anlo anlotinib. **C**, **D** Cell viability assays of H23 (**C**) and H2122SR (**D**) treated with sotorasib alone or in combination with anlotinib after transfecting empty vector (EV) or MYC overexpressing (OE) plasmids. Results are shown as mean ± SEM. Sot sotorasib, Anlo anlotinib. **E–H** Colony formation assays showing the long-term growth of sotorasib alone or combination treated H23 (**E**, **F**) and H2122SR (**G**, **H**) after overexpression of EV or MYC. Statistical differences are determined using two-way ANOVA with Tukey’s multiple comparisons test. Bold: *p* < 0.05. *n* = 3 per group. **I–L** Cell apoptosis assays of H23 (**E**, **F**) and H2122SR (**G**, **H**) treated with anlotinib (2 μM) plus sotorasib (1 μM) for 24 h after overexpression of EV or MYC. Results are shown as mean ± SEM. Statistical differences are determined using one-way ANOVA with Tukey’s multiple comparisons test. Bold: *p* < 0.05. *n* = 3 per group.

The combination treatment further downregulated MYC target pathways compared to single-agent treatments (Fig. 3F). qPCR and Western blot analysis confirmed significantly elevated MYC expression in H2122SR and H2030SR compared to parental lines in both mRNA and protein levels (Figs. 4A and S4F), while the combination treatment effectively suppressed the mRNA and protein levels of MYC across all resistant cell lines (Figs. 4B and S4G). Notably, MYC overexpression reduced the sensitivity of resistant cells to sotorasib; however, combining anlotinib with sotorasib effectively reversed the impact of MYC overexpression on cell viability (Fig. 4C, D), and long-term survival (Fig. 4E–H). And MYC overexpression partially rescued cells from the combination treatment’s inhibitory effects on cell viability (Fig. S4H), long-term survival (Fig. 4E–H), and apoptosis (Fig. 4I–L).

### c-Myc/ORC2 axis is the crucial signaling pathway underlying the response to anlotinib and KRAS-G12Ci combined treatment

**Fig. 5:**
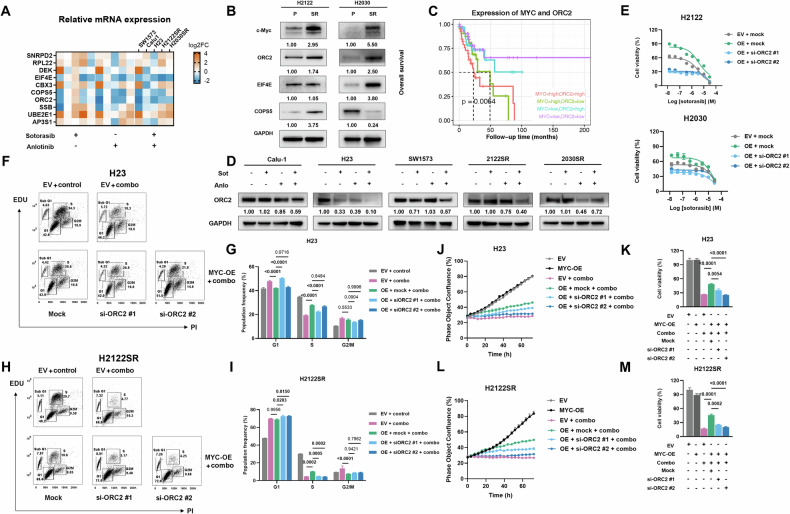
c-Myc/ORC2 axis is the crucial signaling pathway underlying the response to anlotinib and KRAS-G12Ci combined treatment. **A** Heatmap of the mRNA expression of SNRPD2, RPL22, DEK, EIF4E, COPS5, ORC2, SSB, UBE2E1, AP3S1 in five cell lines (Calu-1, SW1573, H23, H2122SR, and H2030SR) detected by qPCR. *n* = 3 per group. **B** The expression of eIF4E, COPS5, and ORC2 in H2122SR and H2030SR compared with parental cell lines detected by western blotting. **C** Survival plot of 154 KRAS-mutant NSCLC patients with different mRNA expression status of MYC and ORC2. Statistical differences are assessed using the log-rank test. Data are from TCGA Pan-Cancer Atlas. **D** The expression of ORC2 in five cell lines (Calu-1, SW1573, H23, H2122SR, and H2030SR) treated with anlotinib (2 μM) plus sotorasib (1 μM) for 24 h detected by western blotting. Sot sotorasib, Anlo anlotinib. **E** Cell viability assays of H2122 and H2030 after MYC overexpression and ORC2 knockdown (si-RNA 20 μM, 72 h). Results are shown as mean ± SEM. **F–I** EdU/PI staining assays of H23 (**F**, **G**) and H2122SR (**H**, **I**) treated with anlotinib (2 μM) plus sotorasib (1 μM) for 24 h after transfected with EV, MYC-OE, si-ORC2 #1 or si-ORC2 #2 (20 μM, 72 h). Results are shown as mean ± SEM. Statistical differences are determined using one-way ANOVA with Tukey’s multiple comparisons test. Bold: *p* < 0.05. *n* = 3 per group. Combo: anlotinib plus sotorasib. **J–M** Cell growth and cell viability assays of H23 (**J**, **K**) and H2122SR (**L**, M) treated with anlotinib (2 μM) plus sotorasib (1 μM) for 24 h EV, MYC-OE, si-ORC2 #1 or si-ORC2 #2 (20 μM, 72 h). Results are shown as mean ± SEM. Statistical differences are determined using one-way ANOVA with Tukey’s multiple comparisons test. Bold: *p* < 0.05. *n* = 3 per group. Combo: anlotinib plus sotorasib.

To identify key downstream effectors of c-Myc in mediating combination treatment response, we analyzed the top 10 most downregulated genes in the MYC targets pathway. qPCR validation across resistant cell lines revealed that EIF4E, COPS5, and ORC2 were consistently downregulated after 24 h of combination treatment (Fig. 5A). While MYC overexpression upregulated all three targets at the mRNA level (Fig. S4I), only ORC2 expression was elevated in H2122SR and H2030SR compared to parental lines (Fig. 5B). Proteasome inhibition experiments revealed that MG132 stabilized c-Myc protein expression. Furthermore, the inhibitory effects of anlotinib, either alone or in combination with sotorasib, were partially reversed, indicating that these effects were mediated, at least in part, by a reduction in protein stability as well (Fig. S4J).

Analysis of TCGA datasets (154 KRAS-mutant NSCLC patients) revealed that high MYC expression correlated with poor overall survival (Fig. S4K) [19]. Notably, ORC2 expression positively correlated with MYC expression (Fig. S4L), and patients with high co-expression of both genes showed the worst survival outcomes (Fig. 5C).

ORC2, as part of the DNA replication initiator complex, functions alongside other ORC subunits (ORC1-6) to mediate DNA replication, playing a crucial role in carcinogenesis [20]. As expected, combination treatment suppressed the protein expression of ORC2 (Fig. 5D). ORC2 knockdown (Fig. S5A) restored sotorasib sensitivity from MYC-OE-induced resistance in H2122 and H2030 parental cell lines (Fig. 5E). Overexpression of MYC upregulated ORC2 in mRNA and protein levels (Figs. S4I and S5A), and ORC2 knockdown reversed the protective effects of MYC overexpression against combination treatment, restoring cell cycle arrest and apoptosis in H23 and H2122SR cells (Figs. 5F–I, and S5B–E). Similarly, ORC2 knockdown reinstated the combination treatment’s inhibitory effects on tumor growth and viability in MYC-overexpressing cells (Fig. 5J–M).

These findings establish the c-Myc/ORC2 axis as a critical mediator of response to anlotinib combined with KRAS-G12Ci in overcoming resistance.

### Anlotinib combined with KRAS-G12Ci exerts potent tumor suppression in resistant settings in vivo

**Fig. 6:**
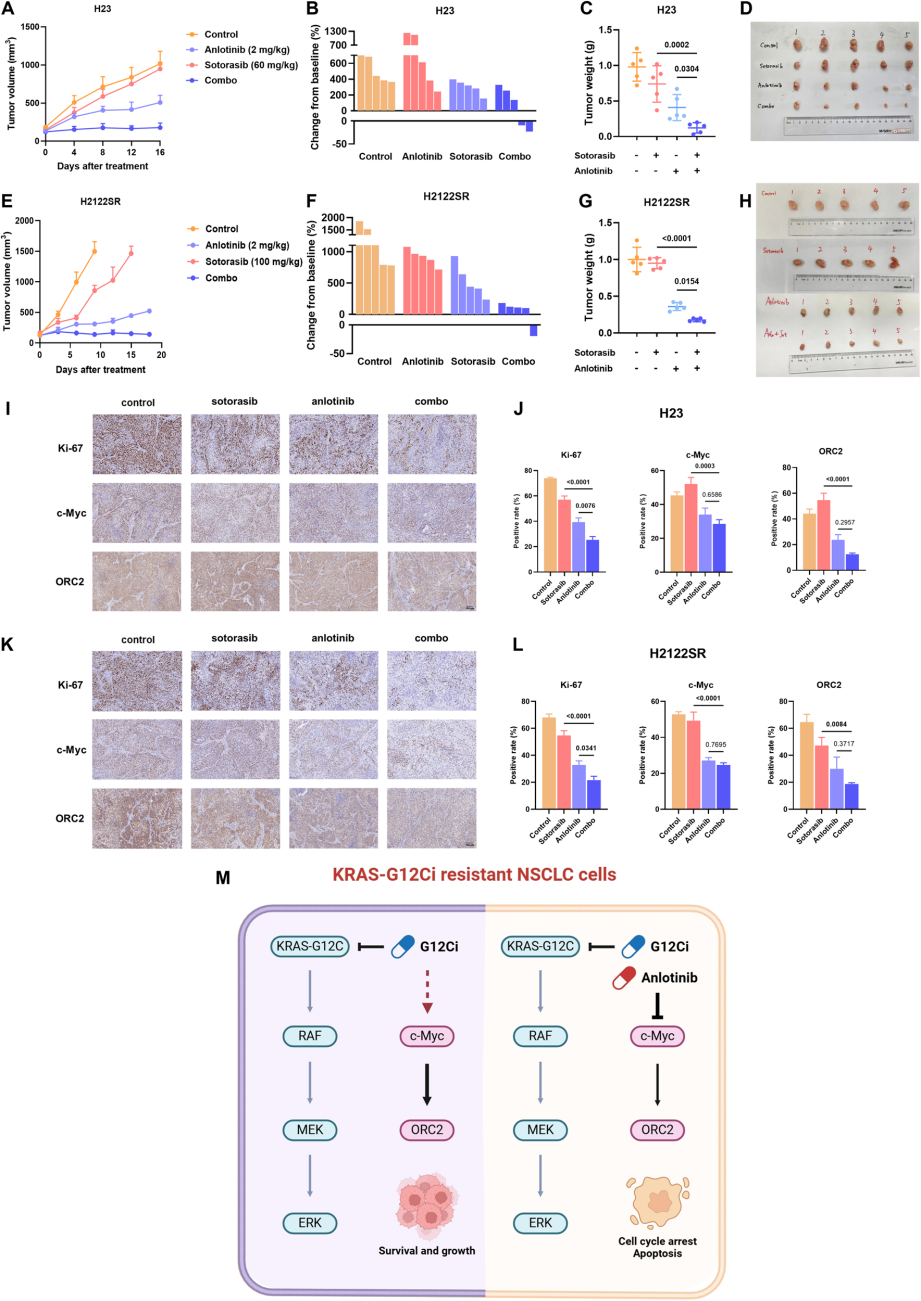
Anlotinib combined with KRAS-G12Ci exerts potent tumor suppression in resistant settings in vivo. **A–H** The antitumor effects of anlotinib combined with sotorasib in H23 CDX model (**A**–**D**) and H2122SR CDX model (**E**–**H**). Results are shown as mean ± SEM. Statistical differences are determined using one-way ANOVA with Tukey’s multiple comparisons test. Bold: *p* < 0.05. *n* = 5 per group. Combo: anlotinib plus sotorasib. **I–L** The expression of Ki-67, c-Myc, and ORC2 in H23 CDX model (**I**, **J**) and H2122SR CDX model (**K**, **L**) detected by IHC. Results are shown as mean ± SEM. Statistical differences are determined using one-way ANOVA with Tukey’s multiple comparisons test. Bold: *p* < 0.05. *n* = 5 per group. Combo: anlotinib plus sotorasib. **M** Graphic abstract of c-Myc/ORC2 inhibition induced by anlotinib plus sotorasib in KRAS-G12Ci resistant NSCLC cells. Created with Biorender.com.

To validate our in vitro findings, we evaluated the combination treatment’s efficacy in vivo using H23 and H2122SR CDX models. While single-agent sotorasib showed limited efficacy, the combination treatment demonstrated potent tumor growth suppression without significant effects on body weight in both models (Figs. 6A–H and S5F, G).

Immunohistochemical analysis of tumor tissues revealed that the combination treatment significantly decreased the expression of Ki-67 (proliferation marker), c-Myc, and ORC2 in both H23 and H2122SR xenografts (Fig. 6I–L).

Supporting these findings, TMA analysis demonstrated a positive correlation between ORC2 and c-Myc protein expression levels. Importantly, high co-expression of c-Myc and ORC2 correlated with worse survival outcomes in NSCLC patients (Fig. S5H, I).

These in vivo results along with in vitro results validate our mechanistic findings and demonstrate that anlotinib combined with KRAS-G12Ci effectively overcomes resistance through modulation of the c-Myc/ORC2 axis, resulting in significant tumor suppression (Fig. 6M).

## Discussion

The emergence of KRAS-G12C inhibitors represents a significant breakthrough in NSCLC treatment, yet their clinical efficacy remains constrained by primary and acquired resistance mechanisms [9, 10, 12]. Our study demonstrates that combining anlotinib with KRAS-G12C inhibitors effectively overcomes these resistance mechanisms through modulation of the c-Myc/ORC2 signaling axis.

Our findings reveal that anlotinib synergistically enhances the antitumor effects of KRAS-G12Ci across multiple resistant NSCLC cell lines. This synergy is observed in both primary resistant lines (H23, Calu-1, SW1573) and acquired resistant lines (H2122SR, H2030SR), suggesting a broad applicability of this combination strategy. The enhanced efficacy is manifested through increased inhibition of cell viability, long-term survival, and migration, indicating a multifaceted impact on cancer cell behavior. Mechanistically, the combination therapy induces robust cell cycle arrest and apoptosis in resistant cell lines.

Transcriptome analysis and further validation revealed the c-Myc/ORC2 axis as a critical mediator of the combination therapy’s effects. The downregulation of c-Myc and its downstream target ORC2 appears to be a key event in the antitumor response. As part of the DNA replication initiator complex, ORC2 functions alongside other ORC subunits (ORC1-6) to mediate DNA replication, playing a crucial role in carcinogenesis [20]. Interestingly, a study in pancreatic cancer discovered that ORC2 phosphorylation by polo-like kinase 1 promotes DNA replication under stress conditions and contributes to gemcitabine resistance, suggesting its potential importance in cancer progression [21]. Consistent with our in vitro results, the positive correlation between c-Myc and ORC2 expression, and their association with worse survival outcomes in NSCLC patients, further underscores the clinical relevance of targeting this axis. Previous studies have confirmed the importance of upregulated MYC in KRAS-G12Ci resistance and the potential of targeting c-Myc to improve KRAS-G12Ci efficacy [11, 22]. Anlotinib has been shown to directly target c-Myc in multiple myeloma [23], and reverse resistance through targeting the c-MET/MYC/AXL axis in NSCLC [24]. These findings strengthen our study’s results of the inhibitory effects of anlotinib combined with KRAS-G12Ci by targeting c-Myc/ORC2 axis in KRAS-G12Ci resistant settings. Clinical responses to anlotinib combination treatment in KRAS-mutant patients have been reported in multiple cancer types, especially NSCLC [25–28], providing evidence for the potential application of our combined strategy.

The in vivo studies corroborate our in vitro findings, demonstrating potent tumor suppression in both primary resistant (H23) and acquired resistant (H2122SR) xenograft models. The significant reduction in tumor growth, coupled with decreased expression of Ki-67, c-Myc, and ORC2 in tumor tissues, provides the evidence for the translational potential of this combination therapy.

Anlotinib has been approved by the NMPA since 2018 as a third-line or subsequent treatment option for patients with NSCLC in China, demonstrating a favorable safety profile as monotherapy in the ALTER0303 trial. Real-world studies have further confirmed that adverse events associated with anlotinib, whether administered alone or in combination with PD-1 inhibitors, are manageable in lung cancer and other malignancies [29–32]. Additionally, multiple clinical trials have demonstrated anlotinib’s potential as a safe and effective candidate when combined with PD-1 inhibitors or EGFR-TKIs, significantly prolonging PFS and overcoming resistance in patients with metastatic NSCLC [33–35]. These findings further support the rationale for exploring anlotinib in combination with KRAS-G12C inhibitors as a promising therapeutic strategy for this patient population.

However, several limitations and future directions should be considered. While our study focused on NSCLC, the applicability of this combination strategy to other KRAS-G12C mutant cancers requires further investigation. Additionally, long-term safety and efficacy of this combination in patients need evaluation through clinical trials. Moreover, the mechanism underlying c-Myc inhibition by this combination necessitates clarification. Future studies should explore potential resistance mechanisms to this combination therapy and strategies to overcome them.

In conclusion, this study provides a strong rationale for the clinical evaluation of anlotinib in combination with KRAS-G12C inhibitors in NSCLC patients with primary or acquired resistance to KRAS-G12Ci monotherapy. The elucidation of the c-Myc/ORC2 axis as a key mediator of response offers new insights into the molecular basis of KRAS-G12Ci resistance and suggests novel therapeutic targets. This combination strategy holds promise for improving outcomes in patients with KRAS-G12C mutant NSCLC, potentially extending the benefits of precision medicine to a broader patient population.

## Supplementary information

Supplementary Figure Legends

Supplementary Figures

Supplementary Table 1

Original Western Blot

## Data Availability

RNA-seq data has been deposited to CNCB-NGDC database (HRA010830). Data from this study are available upon reasonable request to the corresponding author.
