# Supplementary material for: Anlotinib enhances the efficacy of KRAS-G12C inhibitors through c-Myc/ORC2 axis inhibition in non-small cell lung cancer
Source: Cell Death Dis. 2025 May 2;16(1):356. doi: 10.1038/s41419-025-07687-w (PMC12048666; doi:10.1038/s41419-025-07687-w)
Supplement: Supplementary file 1 — Supplementary Figure Legends [file 41419_2025_7687_MOESM1_ESM.docx]

**Figure S1. Establishment of acquired KRAS-G12Ci resistant cell lines.**

**(A)** Cell viability assays of six KRAS-G12C mutant NSCLC cell lines (Calu-1, SW1573, H23, H358, H2122, and H2030) treated with sotorasib.

**(B)** Workflow of establishing acquire resistant H2122 and H2030 cell lines. Created with Biorender.com.

**(C, D)** Cell viability assays verifying acquire resistance of H2122SR and H2030SR.

**(E)** Cell viability assays of five cell lines (Calu-1, SW1573, H23, H2122SR, and H2030SR) treated with anlotinib.

**(F)** Cell viability assays of parental and resistant H2122 and H2030 treated with anlotinib. Results are shown as mean ± SEM.

**Figure S2. Anlotinib combined with KRAS-G12Ci induces cell cycle arrest and apoptosis in primary and acquired resistant cells.**

**(A, B)** Synergy analysis of anlotinib combined with sotorasib using ZIP, HSA, Bliss, and Loewe models in H23 and H2122SR.

**(C-E)** Cell viability and combination index of anlotinib combined with sotorasib treated Calu-1 (C) SW1573 (D), and H2030SR (E). Results are shown as mean ± SEM. Statistical differences are determined using one-way ANOVA with Tukey’s multiple comparisons test. Bold: p < 0.05. n = 3 per group. CI: combination index; Fa: fraction affected; Sot: sotorasib; Anlo: anlotinib.

**(F-H)** Cell growth of Calu-1 (F) SW1573 (G), and H2030SR (H) treated with anlotinib (4 μM) plus sotorasib (2 μM) monitored using IncuCyte. Results are shown as mean ± SEM. Bold: p < 0.05. n = 3 per group. Combo: anlotinib plus sotorasib.

**(I, J)** Cell viability assays of anlotinib combined with adagrasib treated Calu-1 SW1573, H23 (I), and H2122SR and H2030SR (J). Anlo: anlotinib.

**(K, L)** Colony formation assays of Calu-1, SW1573, and H2030SR treated with anlotinib (2 μM) plus sotorasib (1 μM) for 14 days. Results are shown as mean ± SEM. Statistical differences are determined using one-way ANOVA with Tukey’s multiple comparisons test. Bold: p < 0.05. n = 3 per group. Combo: anlotinib plus sotorasib.

**(M-P)** Wound healing assays of Calu-1 (M, N) and H2122SR (O, P) treated with anlotinib (4 μM) plus sotorasib (2 μM) for 48 hours. Results are shown as mean ± SEM. Statistical differences are determined using two-way ANOVA with Tukey’s multiple comparisons test. Bold: p < 0.05. n = 3 per group. Combo: anlotinib plus sotorasib.

**Figure S3. Anlotinib combined with KRAS-G12Ci induced cell cycle arrest and apoptosis to overcome resistance.**

**(A, B)** EdU / PI staining assays of SW1573 and H2030SR treated with anlotinib (4 μM) plus sotorasib (2 μM) for 24 hours. Results are shown as mean ± SEM. Statistical differences are determined using one-way ANOVA with Tukey’s multiple comparisons test. Bold: p < 0.05. n = 3 per group. Combo: anlotinib plus sotorasib.

**(C, D)** Cell apoptosis assays of SW1573 and H2030SR treated with anlotinib (4 μM) plus sotorasib (2 μM) for 24 and 48 hours. Results are shown as mean ± SEM. Statistical differences are determined using two-way ANOVA with Tukey’s multiple comparisons test. Bold: p < 0.05. n = 3 per group. Combo: anlotinib plus sotorasib.

**Figure S4.** **The sensitization effect of anlotinib on resistant cells is mediated through inhibition of c-Myc.**

**(A)** The expression of phospho-Erk (p-Erk), total Erk (t-Erk), p-Akt, and t-Akt in five cell lines (Calu-1, SW1573, H2122SR, and H2030SR) treated with anlotinib (2 μM) plus sotorasib (1 μM) for 24 hours detected by western blotting.

**(B)** GSEA enrichment plot of MYC targets V1 and MYC targets V2 pathways of primary resistant cell lines compared with sensitive cell lines.

**(C)** Enriched hallmark pathways of relapsed SW837 CDX treated with adagrasib compared with before-treatment. Normalized enrichment score (NES) is plotted. FDR < 0.25 is marked in red. FDR > 0.25 is marked in blue. Data are from GSE225060.

**(D)** Volcano plot showing the transcriptional difference of H2122SR treated with anlotinib (2 μM) plus sotorasib (1 μM) for 24 hours compared with DMSO. DEGs with log_2_FC > 2 and adjusted p value < 0.05 were marked in red. DEGs with log_2_FC < -2 and adjusted p value < 0.05 were marked in blue.

**(E)** GSEA enrichment plot of MYC targets V1 and MYC targets V2 pathways of H2122SR compared with H2122 parental cell line.

**(F)** The mRNA expression of MYC in H2122SR and H2030SR compared with parental cell lines. Results are shown as mean ± SEM.

**(G)** The mRNA expression of MYC in five cell lines after 24h treatment of anlotinib (2 μM) and sotorasib (1 μM). Results are shown as mean ± SEM.

**(H)** Cell viability assays of H23 (C) and H2122SR (D) treated with anlotinib plus sotorasib after transfecting empty vector (EV) or MYC overexpressing (OE) plasmids. Results are shown as mean ± SEM. Sot: sotorasib; Anlo: anlotinib.

**(I)** The mRNA expression of EIF4E, COPS5, and ORC2 of H23 and H2122SR after transfected with EV or MYC-OE plasmids detected by qPCR. Results are shown as mean ± SEM.

**(J)** The protein levels of c-Myc in H23 and H2122SR treated with anlotinib (2 μM) and sotorasib (1 μM) after 6h treatment of MG132 (2 μM) or vehicle control.

**(K)** Survival plot of 154 KRAS-mutant NSCLC patients with different mRNA expression status of MYC. Statistical differences are assessed using the log-rank test. Data are from TCGA Pan-Cancer Atlas.

**(L)** Correlation analysis of MYC and ORC2 expression in 154 KRAS-mutant NSCLC patients. Data are from TCGA Pan-Cancer Atlas.

**Figure S5. c-Myc/ORC2 axis is the crucial signaling pathway underlying the response to anlotinib and KRAS-G12Ci combined treatment.**

**(A)** The expression of c-Myc and ORC2 in H23 and H2122SR after transfected with EV, MYC-OE, si-ORC2 #1 or si-ORC2 #2 (si-RNA 20 μM, 72 hours) detected by western blotting.

**(B-E)** Cell apoptosis assays of H23 (B, C) and H2122SR (D, E) treated with anlotinib (2 μM) plus sotorasib (1 μM) for 24 hours after transfected with EV, MYC-OE, si-ORC2 #1 or si-ORC2 #2 (si-RNA 20 μM, 72 hours). Results are shown as mean ± SEM. Statistical differences are determined using one-way ANOVA with Tukey’s multiple comparisons test. Bold: p < 0.05. n = 3 per group. Combo: anlotinib plus sotorasib.

**(F, G)** Body weight of H23 CDX model and H2122SR CDX model treated with anlotinib combined with sotorasib. Results are shown as mean ± SEM. n = 5 per group. Combo: anlotinib plus sotorasib.

**(H)** Survival plot of 60 NSCLC patients from TMA with either high or low co-expression of c-Myc and ORC2 assessed by IHC. Statistical differences are assessed using the log-rank test.

**(I)** Correlation analysis of MYC and ORC2 expression in 80 NSCLC patients from TMA assessed by IHC.
