# Supplementary material for: Anlotinib enhances the efficacy of KRAS-G12C inhibitors through c-Myc/ORC2 axis inhibition in non-small cell lung cancer
Source: Cell Death Dis. 2025 May 2;16(1):356. doi: 10.1038/s41419-025-07687-w (PMC12048666; doi:10.1038/s41419-025-07687-w)
Supplement: Supplementary file 2 — Supplementary Figures [file 41419_2025_7687_MOESM2_ESM.pdf]

Figure S1

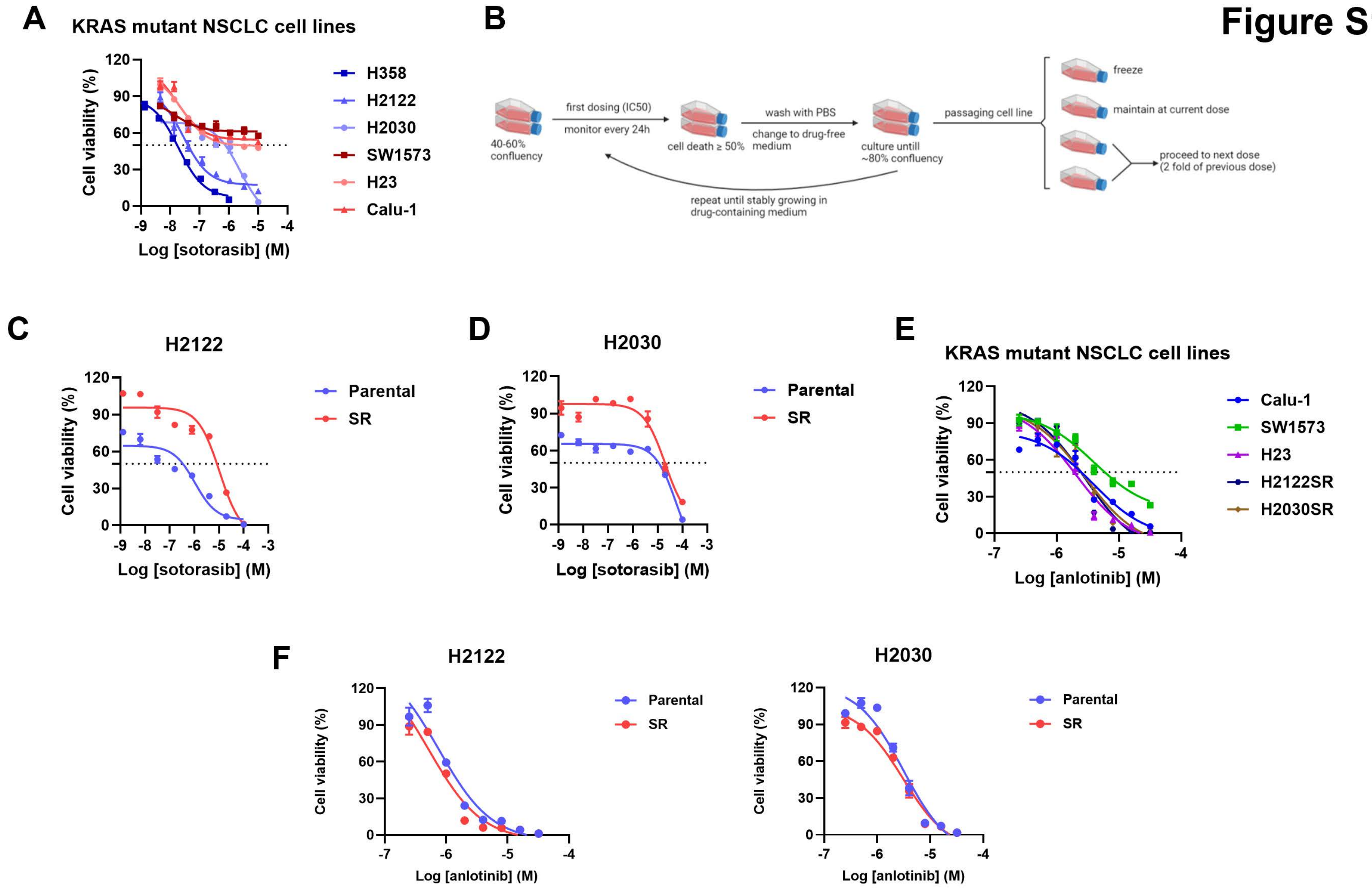
A
KRAS mutant NSCLC cell lines
Cell viability (%)
Log [sotorasib] (M)
H358
H2122
H2030
SW1573
H23
Calu-1
B
first dosing (IC50)
monitor every 24h
40-60% confluency
cell death ≥ 50%
wash with PBS
change to drug-free medium
culture untill ~80% confluency
passaging cell line
repeat until stably growing in drug-containing medium
freeze
maintain at current dose
proceed to next dose (2 fold of previous dose)
C
H2122
Cell viability (%)
Log [sotorasib] (M)
Parental
SR
D
H2030
Cell viability (%)
Log [sotorasib] (M)
Parental
SR
E
KRAS mutant NSCLC cell lines
Cell viability (%)
Log [anlotinib] (M)
Calu-1
SW1573
H23
H2122SR
H2030SR
F
H2122
Cell viability (%)
Log [anlotinib] (M)
Parental
SR
H2030
Cell viability (%)
Log [anlotinib] (M)
Parental
SR

Figure S2

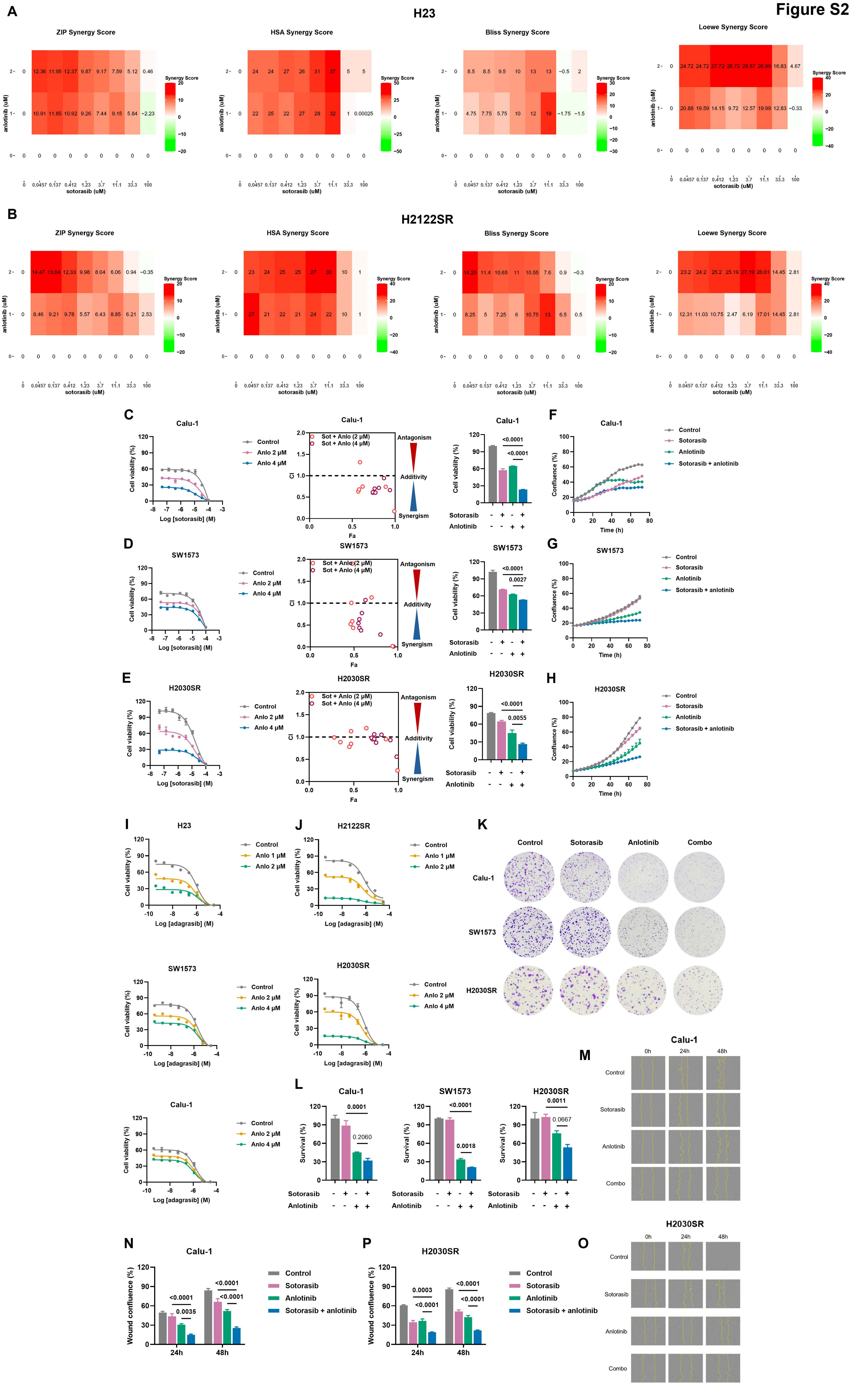
A
H23
ZIP Synergy Score
12.36 11.95 12.37 9.87 9.17 7.59 5.12 0.46
10.91 11.85 10.92 9.26 7.44 9.15 5.84 −2.23
HSA Synergy Score
24 24 27 26 31 37 5 5
22 25 22 27 28 32 1 0.00025
Bliss Synergy Score
8.5 8.5 9.5 10 13 13 −0.5 2
4.75 7.75 5.75 10 12 19 −1.75 −1.5
Loewe Synergy Score
24.72 24.72 27.72 26.72 29.57 26.99 16.83 4.67
20.88 19.59 14.15 9.72 12.57 19.99 12.83 −0.33
Synergy Score
anlotinib (uM)
sotorasib (uM)
0 0.0457 0.137 0.412 1.23 3.7 11.1 33.3 100
B
H2122SR
ZIP Synergy Score
14.47 13.64 12.33 9.98 8.04 6.06 0.94 −0.35
8.46 9.21 9.78 5.57 6.43 8.85 6.21 2.53
HSA Synergy Score
23 24 25 25 27 30 10 1
27 21 22 21 24 22 10 1
Bliss Synergy Score
14.25 11.4 10.65 11 10.55 7.6 0.9 −0.3
8.25 5 7.25 6 10.75 13 6.5 0.5
Loewe Synergy Score
23.2 24.2 25.2 25.19 27.19 26.01 14.45 2.81
12.31 11.03 10.75 2.47 6.19 17.01 14.45 2.81
C
Calu-1
Cell viability (%)
Log [sotorasib] (M)
Control
Anlo 2 μM
Anlo 4 μM
Sot + Anlo (2 μM)
Sot + Anlo (4 μM)
Antagonism
Additivity
Synergism
CI
Fa
<0.0001
<0.0001
Sotorasib
Anlotinib
F
Confluence (%)
Time (h)
Control
Sotorasib
Anlotinib
Sotorasib + anlotinib
D
SW1573
<0.0001
0.0027
G
E
H2030SR
<0.0001
0.0055
H
I
H23
Log [adagrasib] (M)
Control
Anlo 1 μM
Anlo 2 μM
J
H2122SR
SW1573
Anlo 4 μM
Calu-1
K
Control
Sotorasib
Anlotinib
Combo
Calu-1
SW1573
H2030SR
L
Survival (%)
0.0001
0.2060
<0.0001
0.0018
0.0011
0.0667
M
0h
24h
48h
N
Wound confluence (%)
<0.0001
0.0035
<0.0001
<0.0001
P
0.0003
<0.0001
<0.0001
<0.0001
O

Figure S3

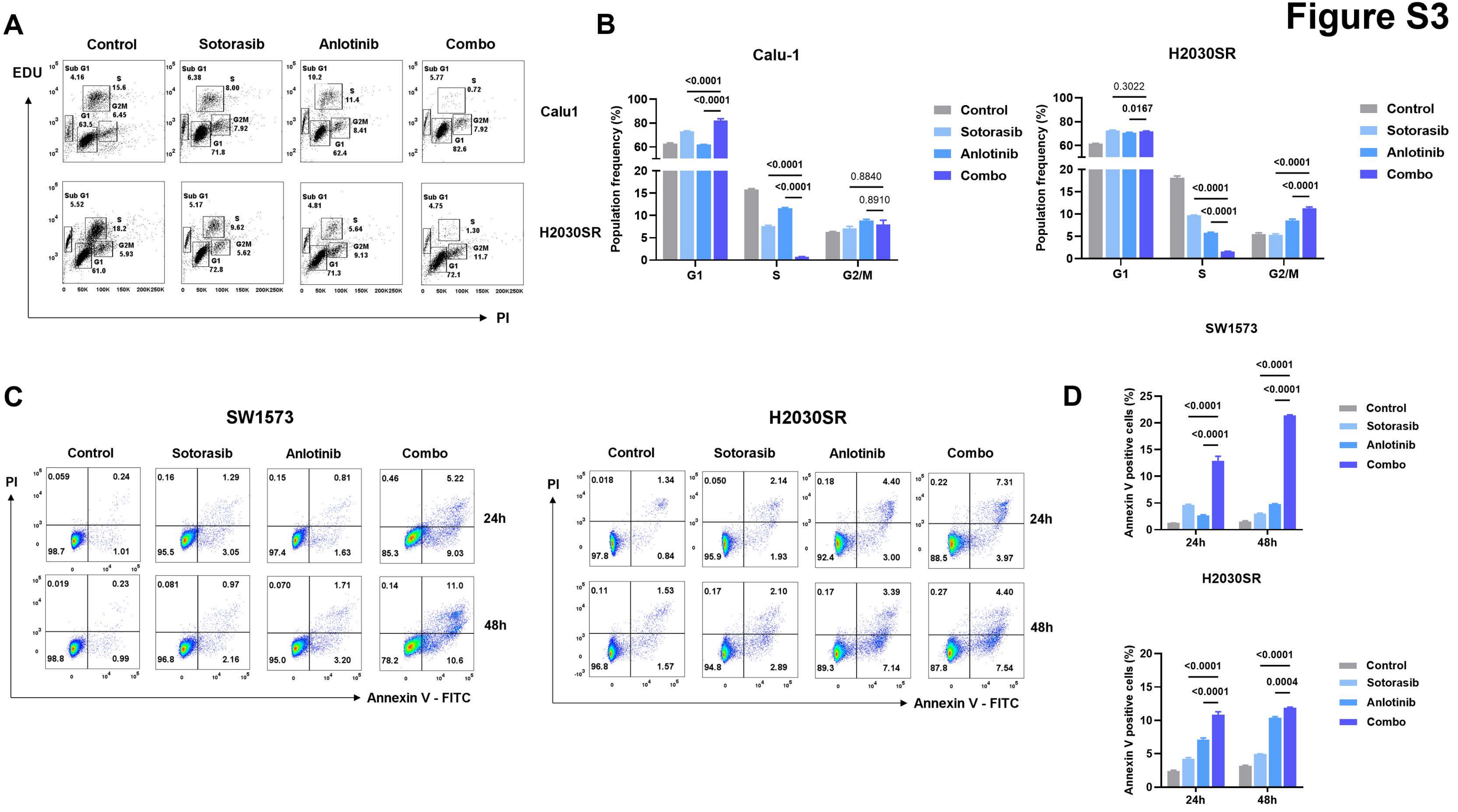

A
Control
Sotorasib
Anlotinib
Combo
EDU
PI
Calu1
H2030SR
Sub G1
S
G1
G2M
B
Calu-1
H2030SR
Population frequency (%)
G1
S
G2/M
Control
Sotorasib
Anlotinib
Combo
<0.0001
0.8840
0.8910
0.3022
0.0167
C
SW1573
H2030SR
24h
48h
Annexin V - FITC
D
SW1573
H2030SR
Annexin V positive cells (%)
0.0004

Figure S4

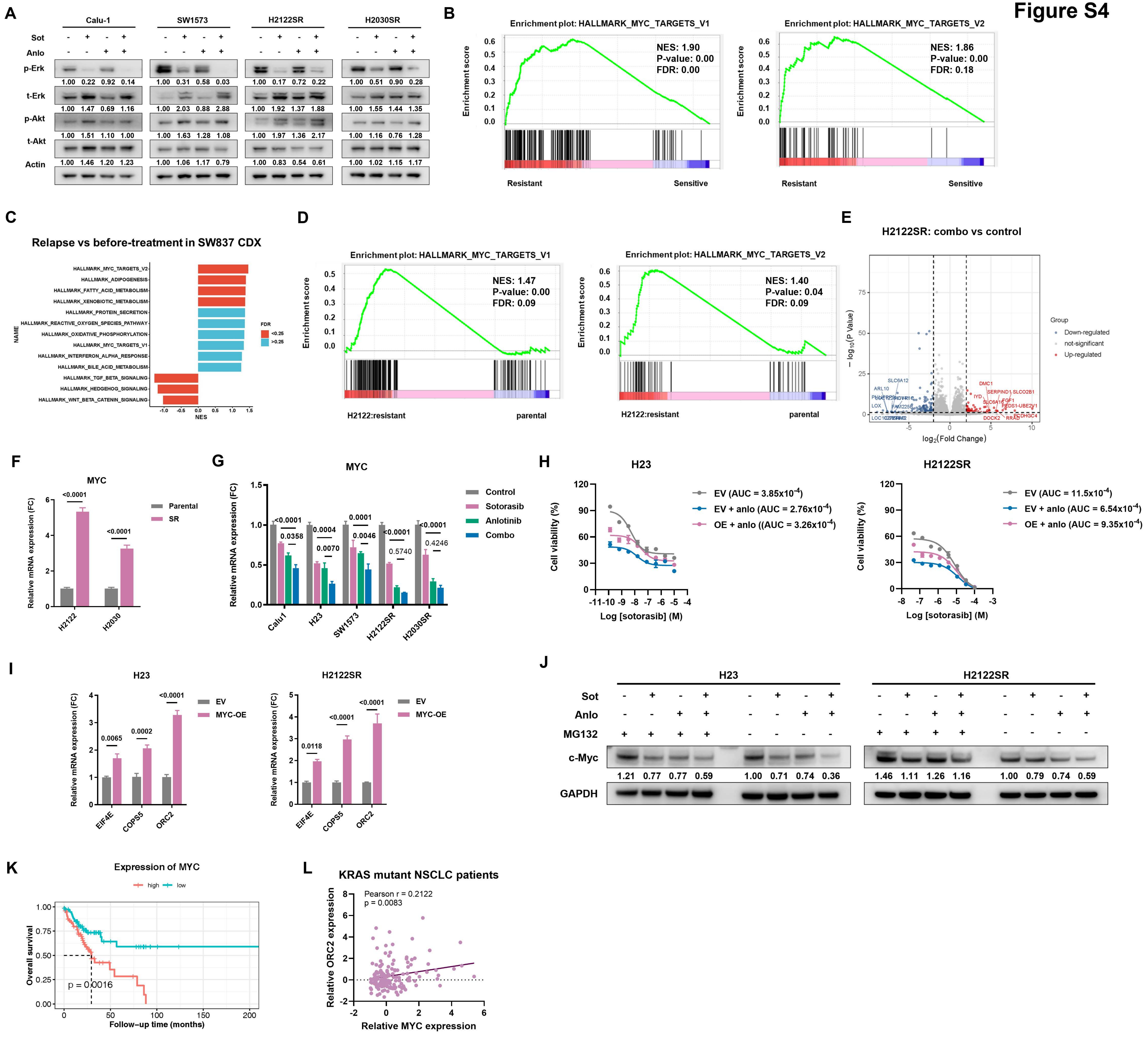
A
Calu-1
SW1573
H2122SR
H2030SR
Sot
Anlo
p-Erk
1.00 0.22 0.92 0.14
1.00 0.31 0.58 0.03
1.00 0.17 0.72 0.22
1.00 0.51 0.90 0.28
t-Erk
1.00 1.47 0.69 1.16
1.00 2.03 0.88 2.88
1.00 1.92 1.37 1.88
1.00 1.55 1.44 1.35
p-Akt
1.00 1.51 1.10 1.00
1.00 1.63 1.28 1.08
1.00 1.97 1.36 2.17
1.00 1.16 0.76 1.28
t-Akt
1.00 1.46 1.20 1.23
1.00 1.06 1.17 0.79
1.00 0.83 0.54 0.61
1.00 1.02 1.15 1.17
Actin
B
Enrichment plot: HALLMARK_MYC_TARGETS_V1
NES: 1.90
P-value: 0.00
FDR: 0.00
Enrichment plot: HALLMARK_MYC_TARGETS_V2
NES: 1.86
P-value: 0.00
FDR: 0.18
Enrichment score
Resistant
Sensitive
C
Relapse vs before-treatment in SW837 CDX
HALLMARK_MYC_TARGETS_V2
HALLMARK_ADIPOGENESIS
HALLMARK_FATTY_ACID_METABOLISM
HALLMARK_XENOBIOTIC_METABOLISM
HALLMARK_PROTEIN_SECRETION
HALLMARK_REACTIVE_OXYGEN_SPECIES_PATHWAY
HALLMARK_OXIDATIVE_PHOSPHORYLATION
HALLMARK_MYC_TARGETS_V1
HALLMARK_INTERFERON_ALPHA_RESPONSE
HALLMARK_BILE_ACID_METABOLISM
HALLMARK_TGF_BETA_SIGNALING
HALLMARK_HEDGEHOG_SIGNALING
HALLMARK_WNT_BETA_CATENIN_SIGNALING
NAME
NES
FDR
<0.25
>0.25
D
Enrichment plot: HALLMARK_MYC_TARGETS_V1
NES: 1.47
P-value: 0.00
FDR: 0.09
Enrichment plot: HALLMARK_MYC_TARGETS_V2
NES: 1.40
P-value: 0.04
FDR: 0.09
Enrichment score
H2122:resistant
parental
E
H2122SR: combo vs control
Group
Down-regulated
not-significant
Up-regulated
$-\log_{10}$(P Value)
$\log_2$(Fold Change)
F
MYC
Parental
SR
Relative mRNA expression (FC)
<0.0001
<0.0001
H2122
H2030
G
MYC
Control
Sotorasib
Anlotinib
Combo
Relative mRNA expression (FC)
<0.0001
0.0358
0.0004
0.0070
0.0001
0.0046
<0.0001
0.5740
<0.0001
0.4246
Calu1
H23
SW1573
H2122SR
H2030SR
H
H23
EV (AUC = 3.85x10^-4)
EV + anlo (AUC = 2.76x10^-4)
OE + anlo ((AUC = 3.26x10^-4)
H2122SR
EV (AUC = 11.5x10^-4)
EV + anlo (AUC = 6.54x10^-4)
OE + anlo (AUC = 9.35x10^-4)
Cell viability (%)
Log [sotorasib] (M)
I
H23
H2122SR
EV
MYC-OE
Relative mRNA expression (FC)
0.0065
0.0002
<0.0001
0.0118
<0.0001
<0.0001
EIF4E
COPS5
ORC2
J
H23
H2122SR
Sot
Anlo
MG132
c-Myc
1.21 0.77 0.77 0.59
1.00 0.71 0.74 0.36
1.46 1.11 1.26 1.16
1.00 0.79 0.74 0.59
GAPDH
K
Expression of MYC
high
low
Overall survival
p = 0.0016
Follow-up time (months)
L
KRAS mutant NSCLC patients
Pearson r = 0.2122
p = 0.0083
Relative ORC2 expression
Relative MYC expression

Figure S5

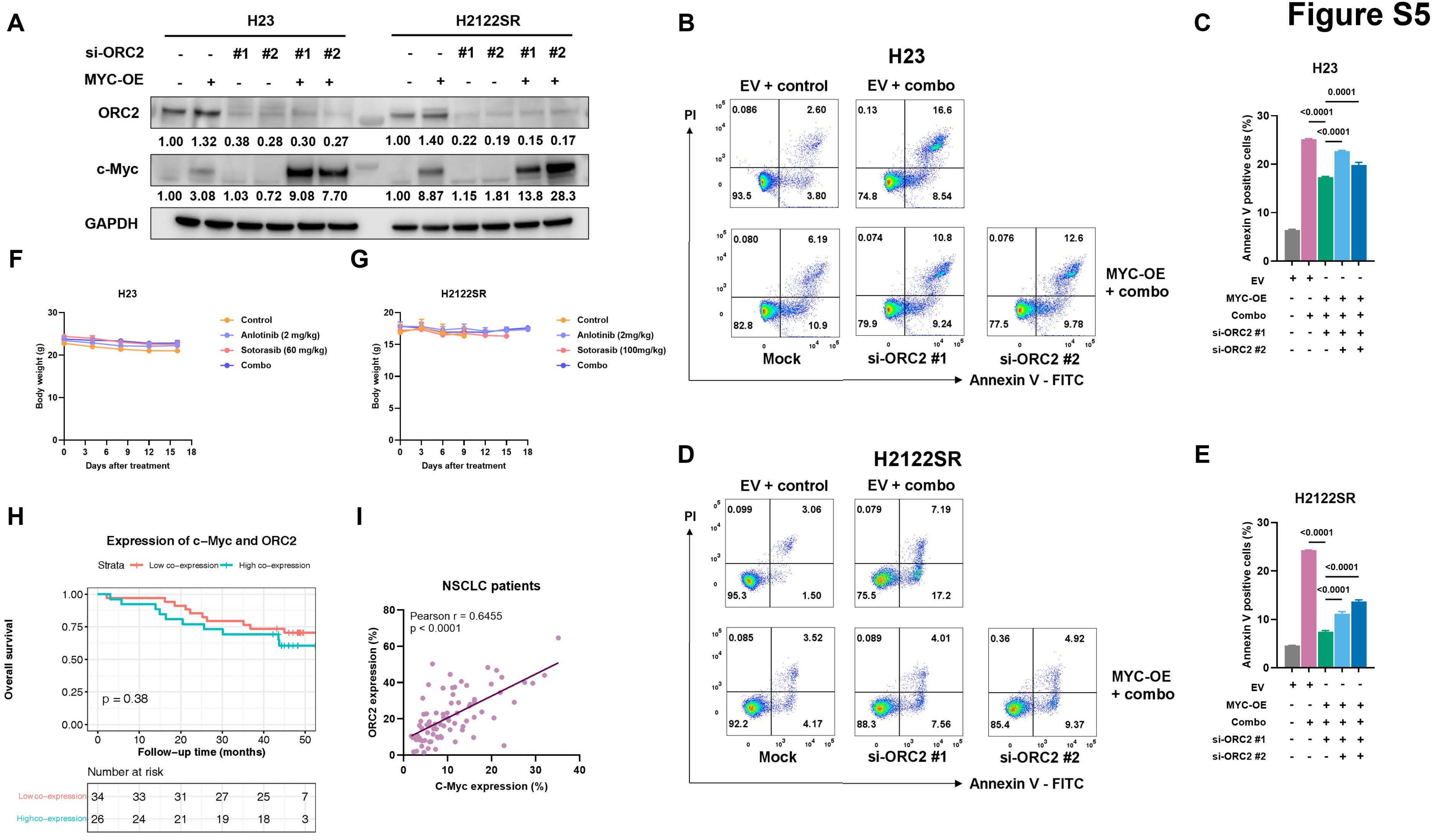

A
H23
H2122SR
si-ORC2 - - #1 #2 #1 #2 - - #1 #2 #1 #2
MYC-OE - + - - + + - + - - + +
ORC2
1.00 1.32 0.38 0.28 0.30 0.27 1.00 1.40 0.22 0.19 0.15 0.17
c-Myc
1.00 3.08 1.03 0.72 9.08 7.70 1.00 8.87 1.15 1.81 13.8 28.3
GAPDH
B
H23
EV + control
EV + combo
PI
0.086 2.60 93.5 3.80
0.13 16.6 74.8 8.54
0.080 6.19 82.8 10.9
0.074 10.8 79.9 9.24
0.076 12.6 77.5 9.78
MYC-OE + combo
Mock
si-ORC2 #1
si-ORC2 #2
Annexin V - FITC
C
H23
Annexin V positive cells (%)
<0.0001
<0.0001
0.0001
EV
MYC-OE
Combo
si-ORC2 #1
si-ORC2 #2
D
H2122SR
EV + control
EV + combo
PI
0.099 3.06 95.3 1.50
0.079 7.19 75.5 17.2
0.085 3.52 92.2 4.17
0.089 4.01 88.3 7.56
0.36 4.92 85.4 9.37
MYC-OE + combo
Mock
si-ORC2 #1
si-ORC2 #2
Annexin V - FITC
E
H2122SR
Annexin V positive cells (%)
<0.0001
<0.0001
<0.0001
EV
MYC-OE
Combo
si-ORC2 #1
si-ORC2 #2
F
H23
Body weight (g)
Days after treatment
Control
Anlotinib (2 mg/kg)
Sotorasib (60 mg/kg)
Combo
G
H2122SR
Body weight (g)
Days after treatment
Control
Anlotinib (2mg/kg)
Sotorasib (100mg/kg)
Combo
H
Expression of c–Myc and ORC2
Strata Low co–expression High co–expression
Overall survival
p = 0.38
Follow–up time (months)
Number at risk
Low co–expression 34 33 31 27 25 7
High co–expression 26 24 21 19 18 3
I
NSCLC patients
Pearson r = 0.6455
p < 0.0001
ORC2 expression (%)
C-Myc expression (%)
