# Supplementary figures and images for: Anlotinib enhances the efficacy of KRAS-G12C inhibitors through c-Myc/ORC2 axis inhibition in non-small cell lung cancer
Source: Cell Death Dis. 2025 May 2;16(1):356. doi: 10.1038/s41419-025-07687-w (PMC12048666; doi:10.1038/s41419-025-07687-w)

Figure 2I

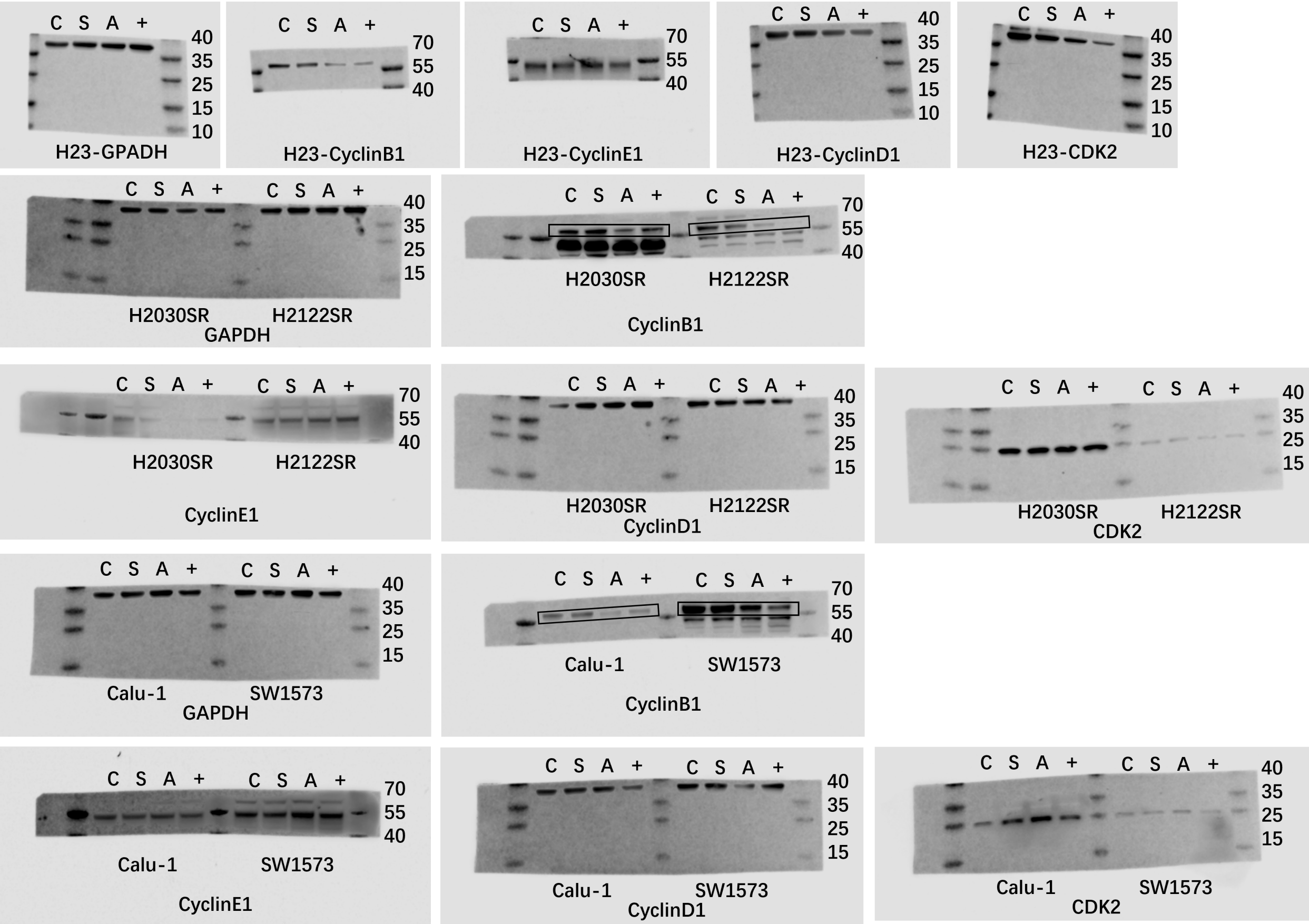

Figure 2J

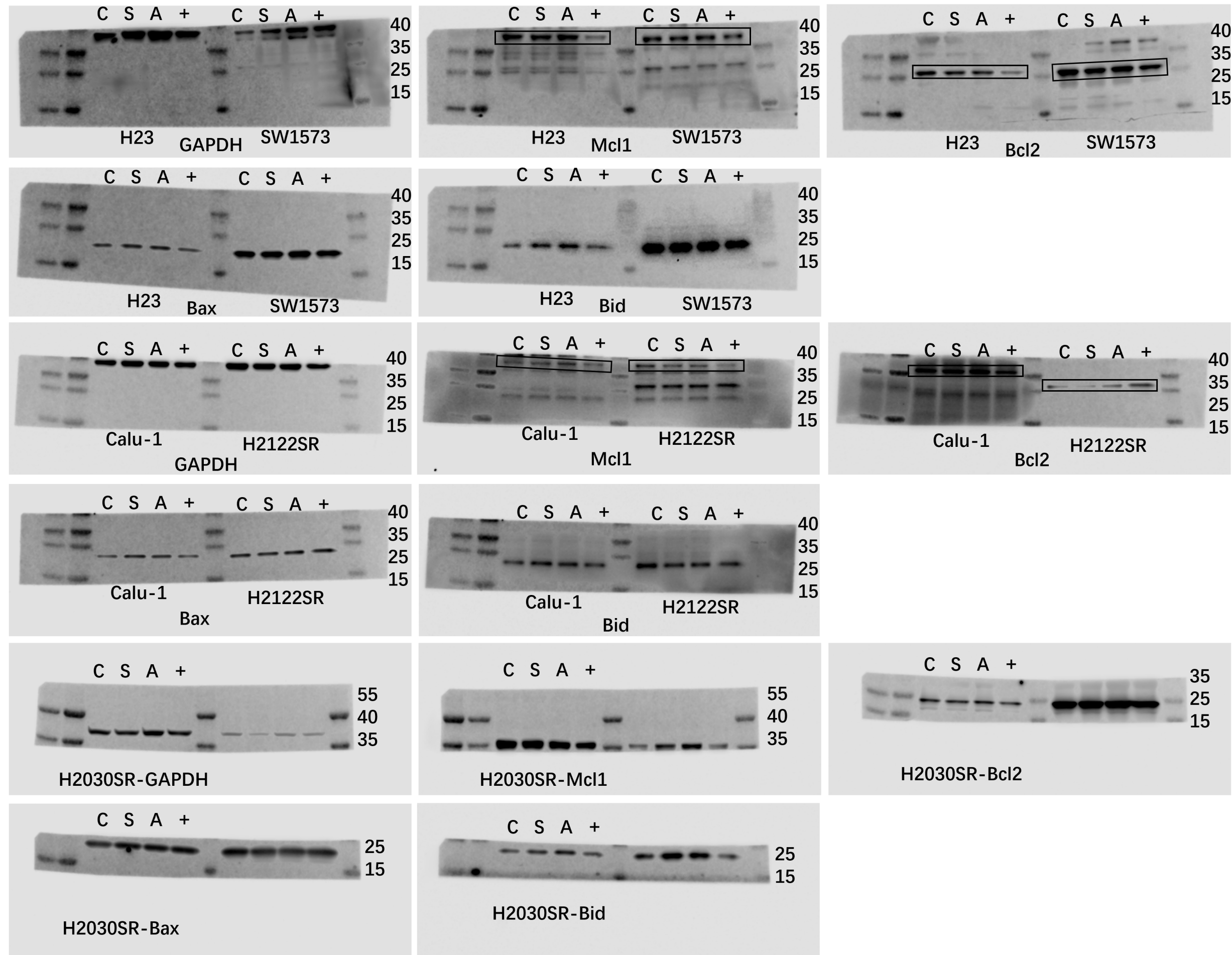

Figure 4A

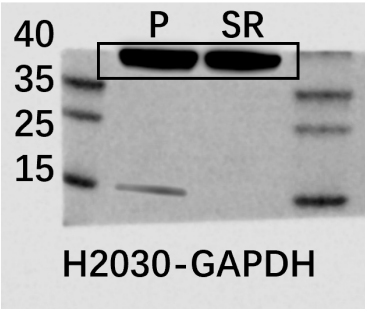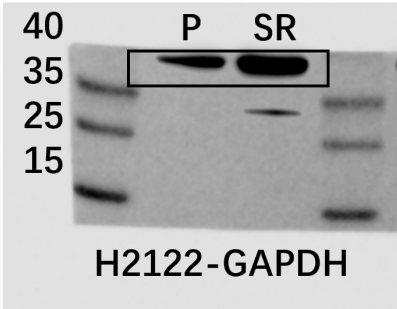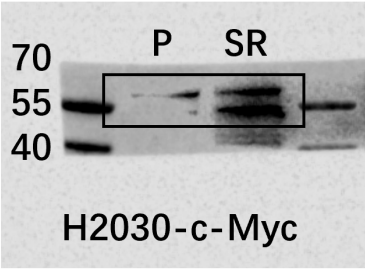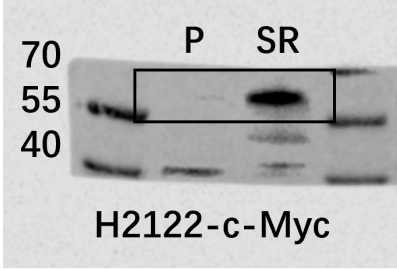

Figure 4B

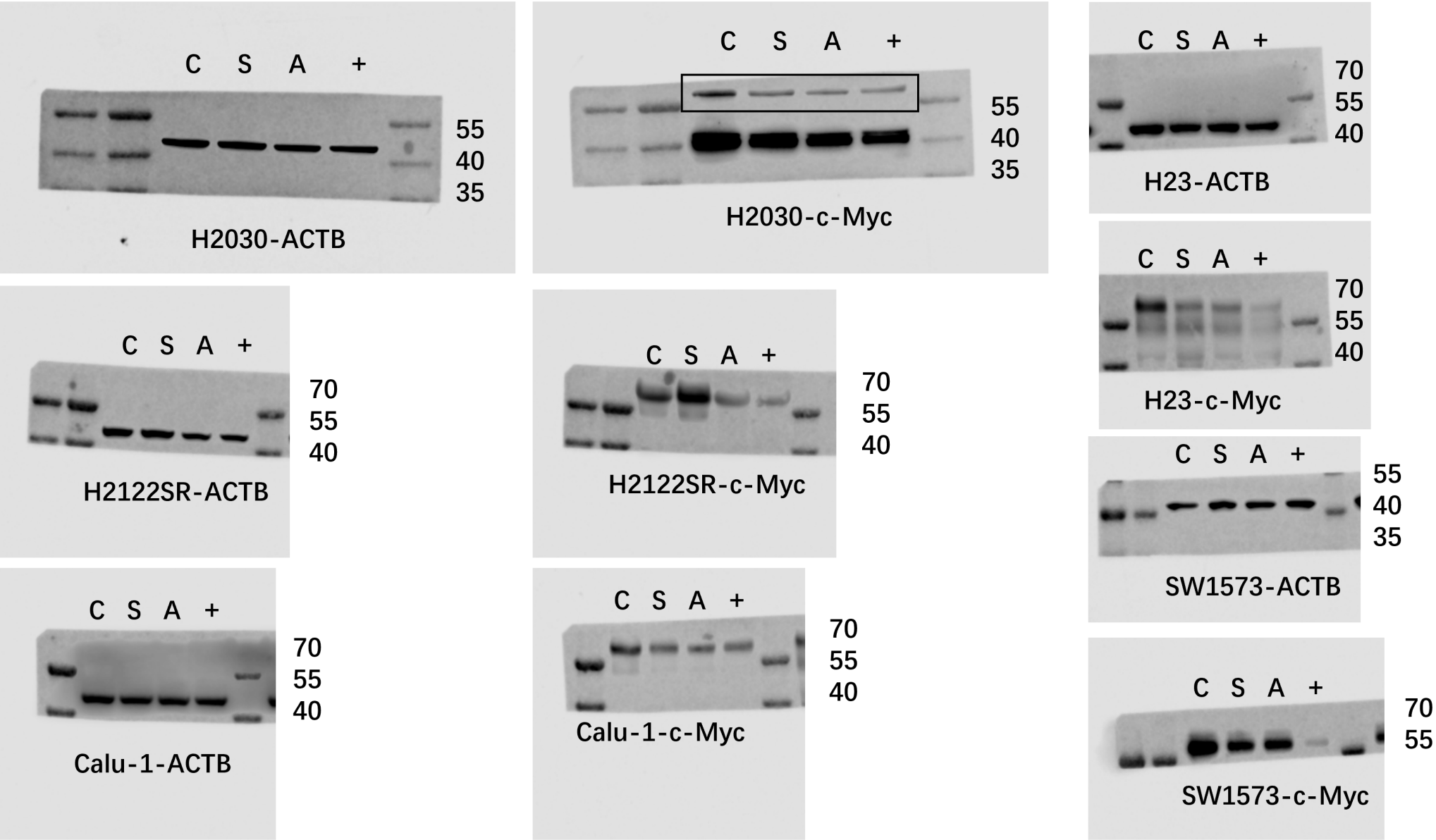

Figure 5B

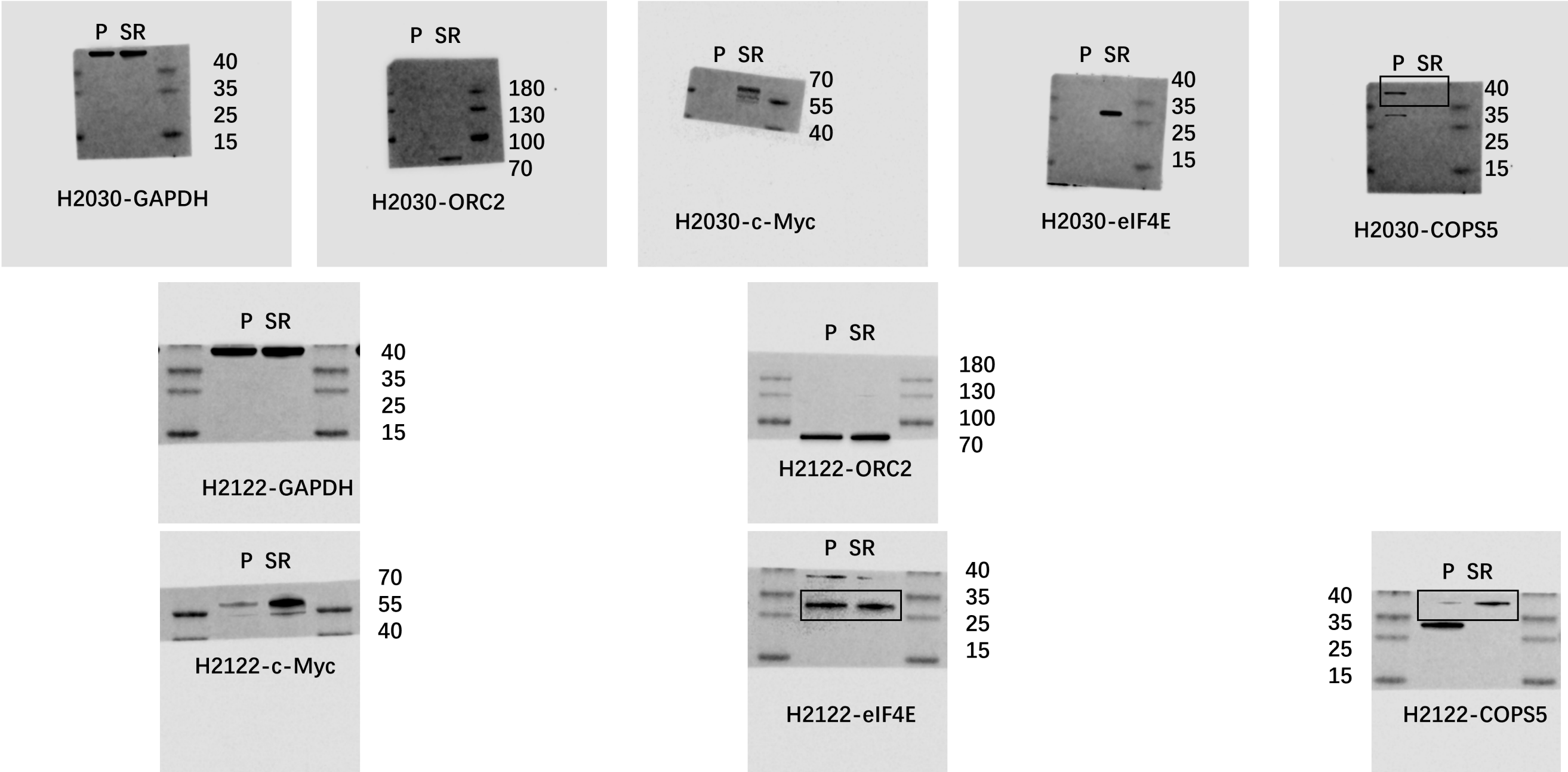

Figure 5D

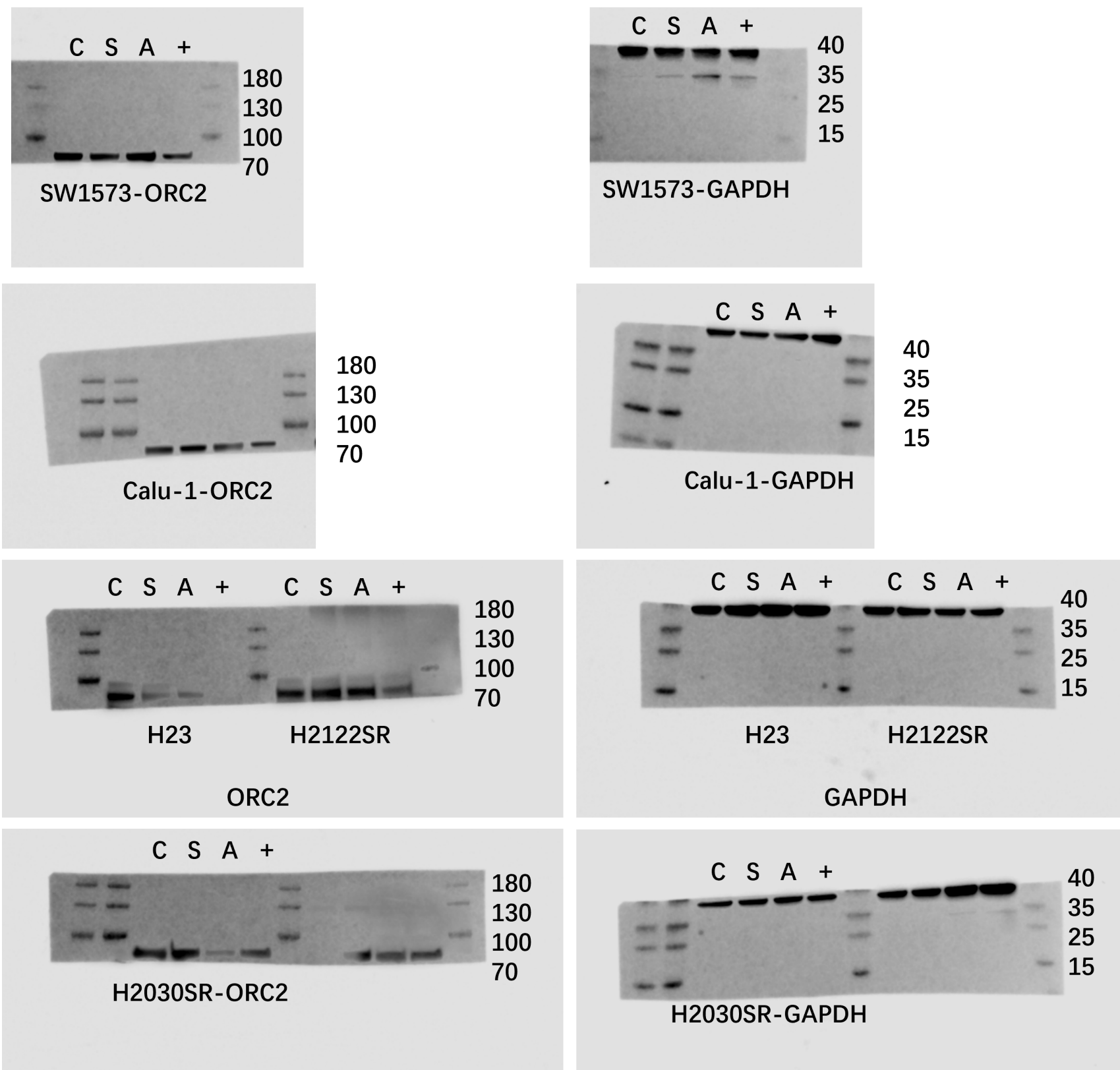

Figure S4A

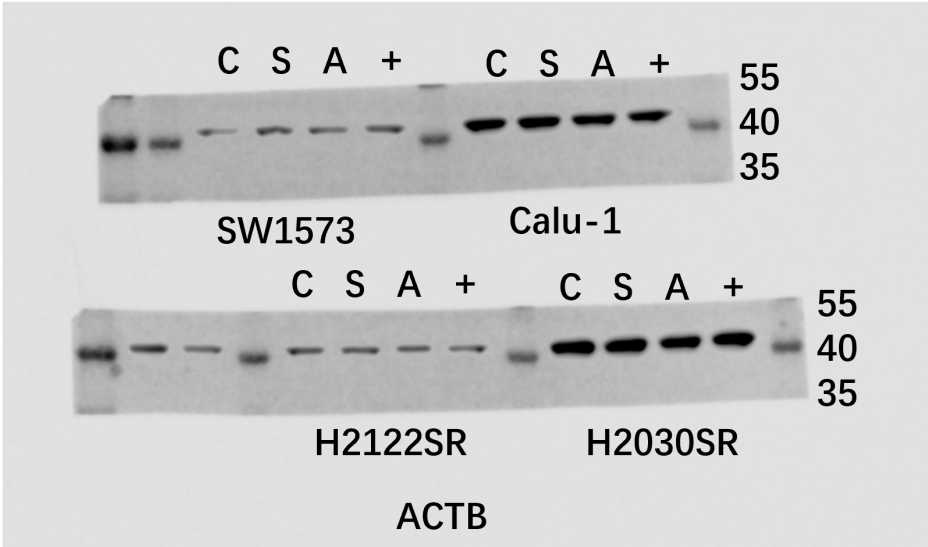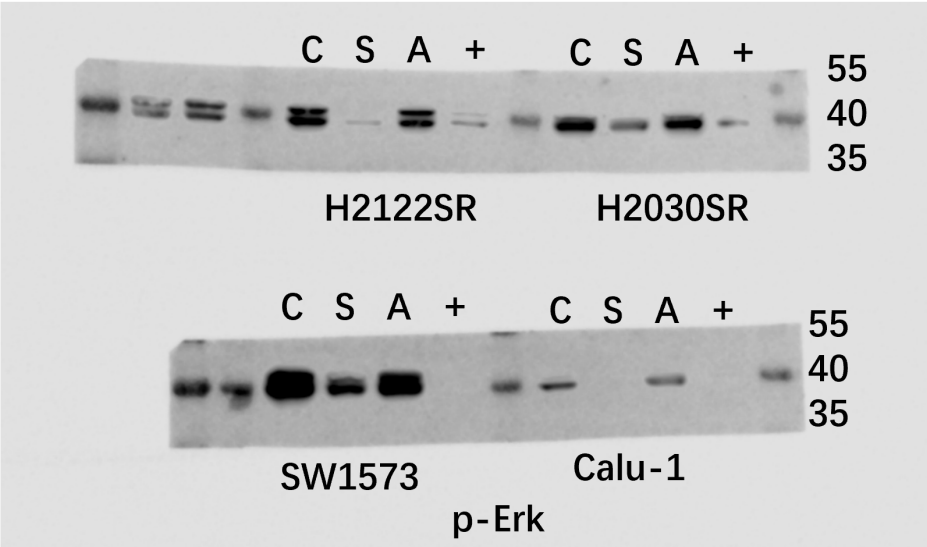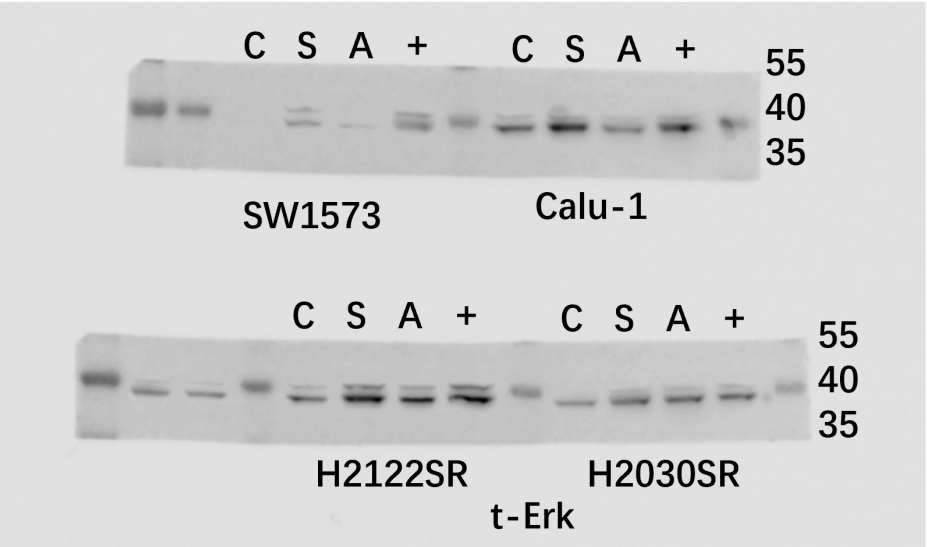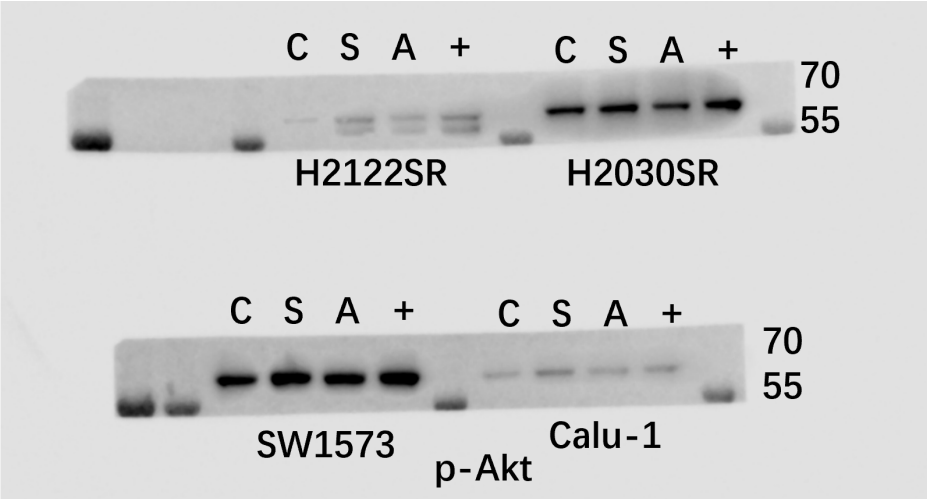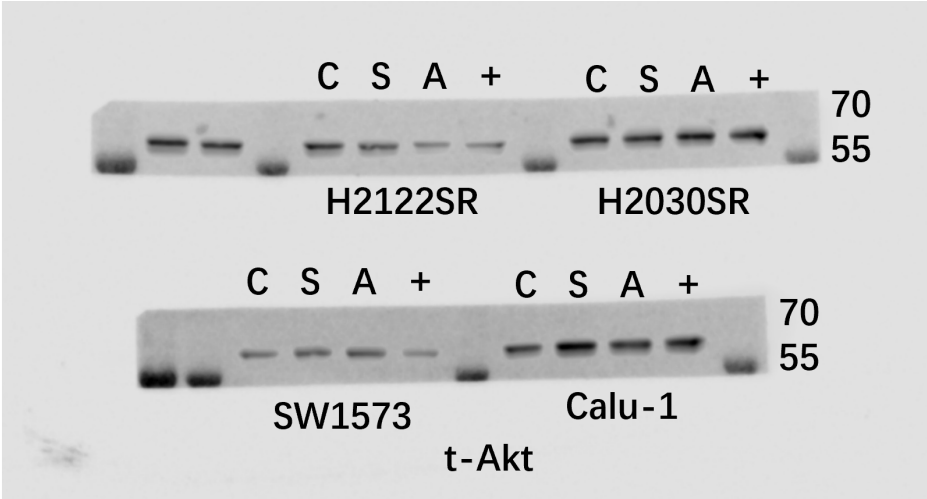

Figure S4J

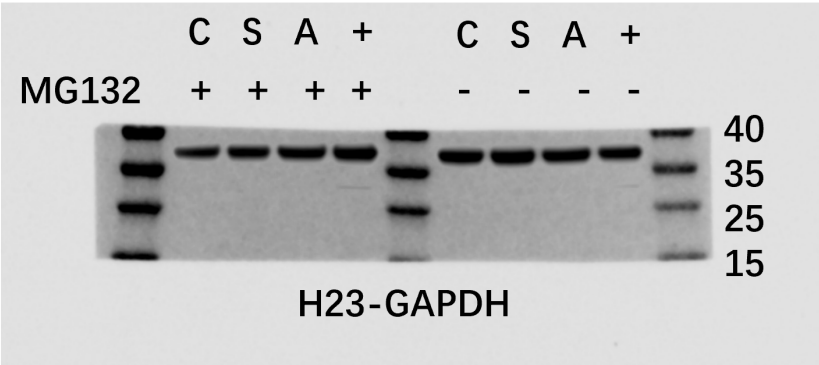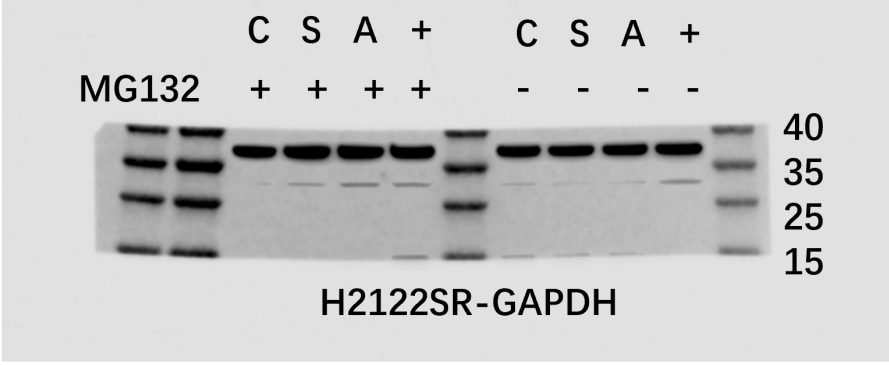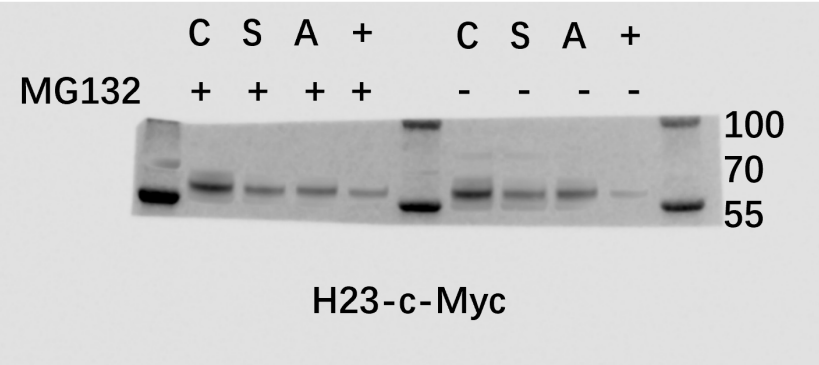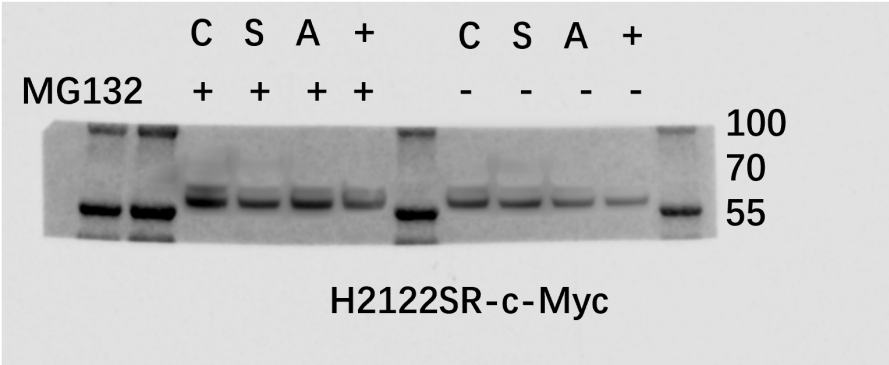

Figure S5A

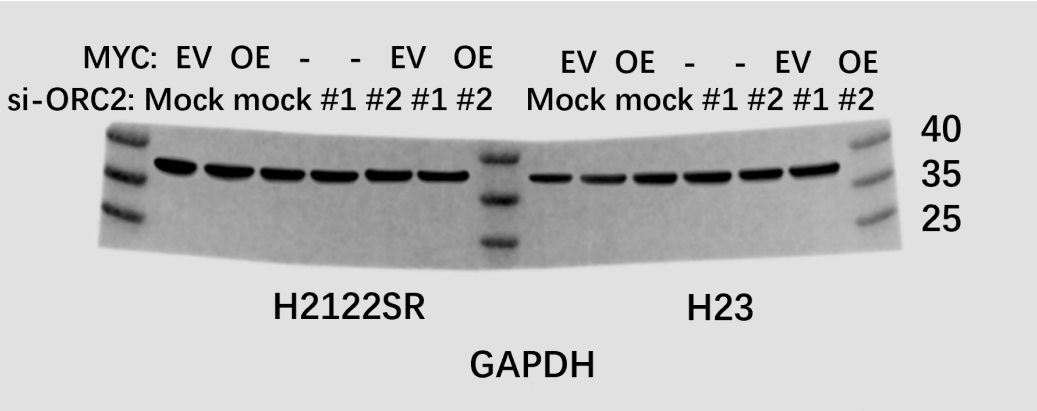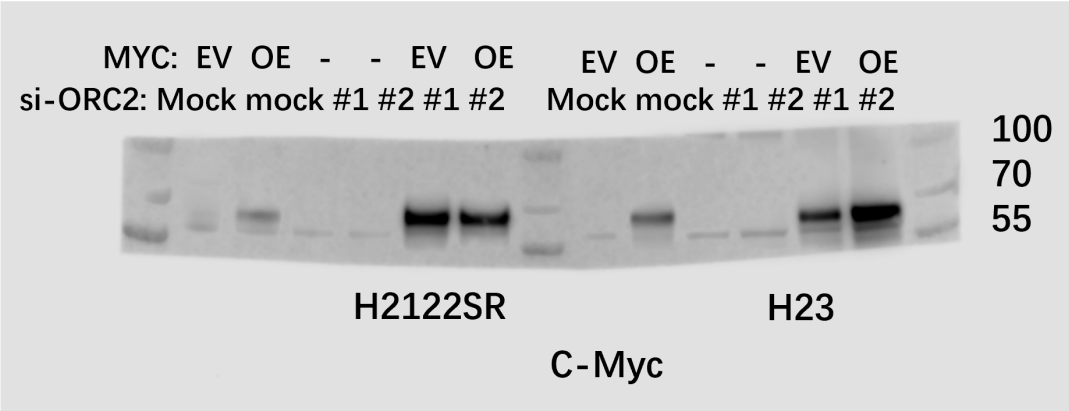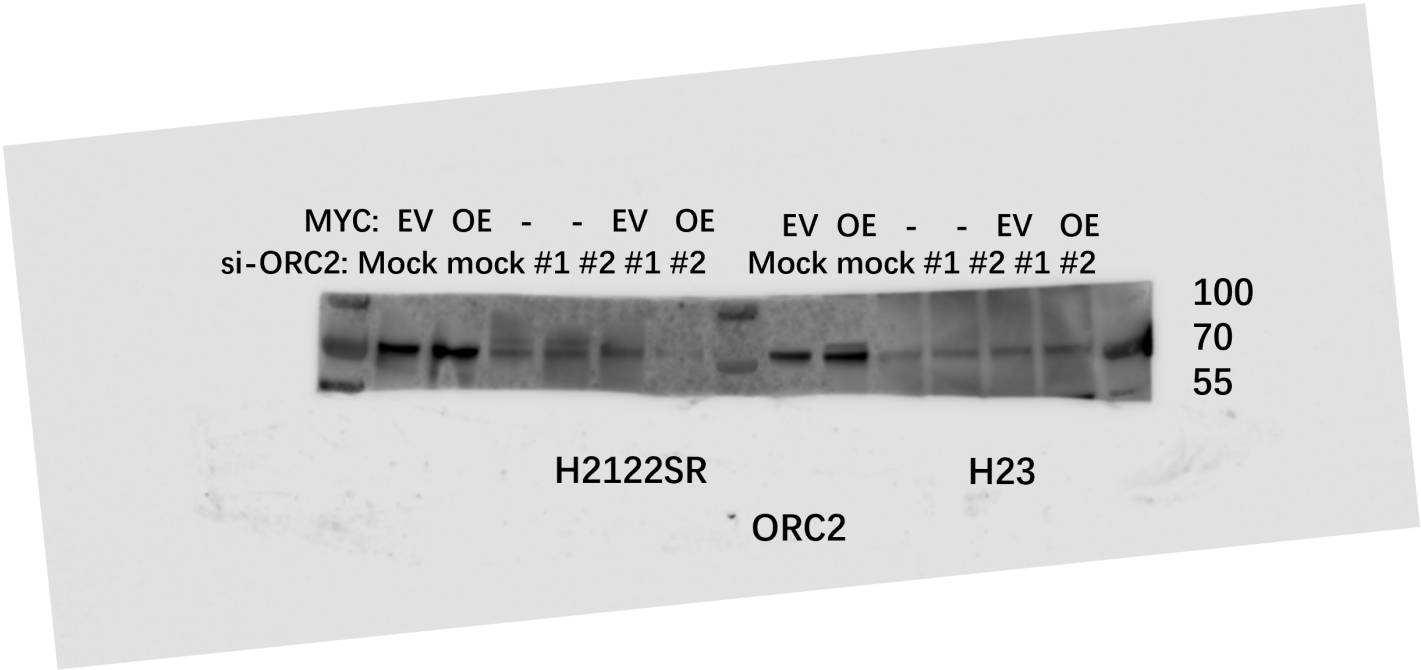

Supplement: Supplementary file 4 — Original Western Blot [file 41419_2025_7687_MOESM4_ESM.pdf]
